# A Dual‐Targeting T6SS DNase Drives Bacterial Antagonism and Eukaryotic Apoptosis via the cGAS‐STING‐TNF Axis

**DOI:** 10.1002/advs.202504086

**Published:** 2025-05-14

**Authors:** Li Song, Lei Xu, Pengfei Zhang, Shuying Li, Yichen Qu, Yixin Zhao, Zhenkun Shi, Ruiqi Ma, Yongdong Li, Yi Chen, Yao Wang, Zhengfan Jiang, Gehong Wei, Xihui Shen

**Affiliations:** ^1^ Shaanxi Key Laboratory of Agricultural and Environmental Microbiology College of Natural Resources and Environment Northwest A&F University Yangling Shaanxi 12100 P. R. China; ^2^ College of Life Sciences Northwest A&F University Yangling Shaanxi 712100 P. R. China; ^3^ Key Laboratory of Cell Proliferation and Differentiation of the Ministry of Education School of Life Sciences Peking University Beijing 100871 P. R. China; ^4^ Peking‐Tsinghua Center for Life Sciences Peking University Beijing 100871 P. R. China; ^5^ Ningbo Municipal Center for Disease Control and Prevention Ningbo Zhejiang 315010 P. R. China

**Keywords:** apoptosis, DNA damage, DNase, trans‐kingdom effector, Type VI secretion systems (T6SSs), *Yersinia pseudotuberculosis*

## Abstract

The Type VI secretion system (T6SS) is a key virulence mechanism utilized by many Gram‐negative bacteria to mediate the microbial competition and host pathogenesis. Despite the identification of diverse T6SS effectors targeting eukaryotic or prokaryotic cells, the trans‐kingdom T6SS effectors that simultaneously target both eukaryotic and prokaryotic cells remain rarely reported. In this study, it is demonstrated that *Yersinia pseudotuberculosis* (*Yptb*) T6SS secretes a DNase effector, TkeA, which induces apoptosis in host cells. The translocation of TkeA into host cells causes nuclear DNA damage. This, in turn, activates the DNA‐sensing cyclic GMP‐AMP synthase (cGAS)/stimulator of interferon genes (STING) pathway. The activation of the cGAS‐STING pathway by TkeA subsequently triggers apoptosis in host cells via extrinsic pathways, with tumor necrosis factor (TNF) signaling playing a critical role. Additionally, TkeA enhances bacterial competition by targeting rival bacteria, thereby promoting host colonization. These findings reveal that the transkingdom T6SS effector TkeA executes a “one weapon, two battlefields” strategy, acting as a trans‐kingdom effector that enhances interbacterial competition while inducing apoptosis in host cells through the activation of the cGAS‐STING‐TNF axis. This highlights a previously unrecognized dimension of bacterial virulence strategies and expands the understanding of host‐pathogen interactions involving T6SS effectors.

## Graphical abstract

The bacterial Type VI secretion system (T6SS) emerges as a sophisticated virulence mechanism, leveraging the dual‐function effector TkeA from *Yersinia pseudotuberculosis*. TkeA concurrently damages rival bacteria and host cell DNA, triggering the host's cGAS‐STING‐TNF pathway and inducing apoptosis. This trans‐kingdom “one weapon, two battlefields” strategy reshapes the understanding of bacterial competition and host‐pathogen interactions.

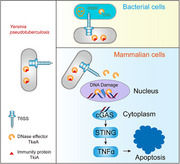

## 1 Introduction

In competitive natural environments with limited resources, bacteria have developed sophisticated survival strategies to outcompete other microorganisms. One such strategy is the type VI secretion system (T6SS), a versatile molecular machine used by many Gram‐negative bacteria to interact with their environment, including host cells and other bacteria.^[^ ^1^, ^2^ ^]^ Resembling an inverted phage tail, the T6SS enables the direct secretion of effectors into target cells or the extracellular environment.^[^ ^2^ ^]^ Although the T6SS apparatus is highly conserved, its secreted effectors exhibit remarkable diversity. The T6SS injects these effectors into both prokaryotic and eukaryotic target cells, making it essential for bacterial survival, competition, and virulence.^[^ ^3^, ^4^, ^5^, ^6^, ^7^, ^8^ ^]^ Among these, trans‐kingdom effectors stand out due to their ability to function in both eukaryotic and prokaryotic cells. Although studies have explored several trans‐kingdom T6SS effectors affecting cytoskeletal dynamics, immune signaling, and organelle structure,^[^ ^9^, ^10^, ^11^ ^]^ these remain limited compared to the larger pool of effectors targeting eukaryotic or prokaryotic cells. Given that DNA is a universal genetic material shared by both eukaryotes and prokaryotes, it is not unexpected that DNase effectors, which have long been recognized as pivotal components in bacterial competition, may possess the capacity to antagonize the eukaryotic cells. This idea is not merely speculative recently, a trans‐kingdom DNase, TafE, secreted by *Acinetobacter baumannii*, was shown to specifically target fungal.^[^ ^12^ ^]^ However, a critical knowledge gap persists: How do these T6SS DNases directly subvert eukaryotic cell fate?

Apoptosis, a form of programmed cell death (PCD), serves as a key guardian by eliminating infected or damaged cells to maintain tissue homeostasis. This non‐inflammatory process prevents the persistence of dysfunctional cells, curbing infection spread and bacterial replication.^[^ ^13^ ^]^ Apoptosis can be initiated by diverse signals through two main pathways. The intrinsic pathway, regulated by the Bcl‐2 protein family, triggers mitochondrial outer membrane permeabilization (MOMP), releasing cytochrome C to activate CASPASE 9, which then engages executioner CASPASE 3/7.^[^ ^14^, ^15^, ^16^ ^]^ The extrinsic pathway, activated by external signals like tumor necrosis factor alpha (TNFα) binding to TNF receptor (TNFR), involves RIPK1 deubiquitylation and phosphorylation, recruiting CASPASE 8 and ultimately activating CASPASE 3.^[^ ^17^, ^18^, ^19^ ^]^ Unlike its typical role in inflammation via MAPK and NF‐κB pathways, TNF signaling can shift toward cell death when inflammation is suppressed.^[^ ^18^ ^]^

DNA damage is a pivotal trigger of apoptosis.^[^ ^20^ ^]^ Pathogenic bacteria often induce DNA damage in host cells, not merely as an infection byproduct, but as a strategic mechanism to manipulate host cell death pathways and enhance bacterial survival.^[^ ^20^, ^21^, ^22^ ^]^ Certain bacteria secrete genotoxic toxins, such as cytolethal distending toxins (CDTs), colibactin, and indolimines.^[^ ^23^, ^24^, ^25^ ^]^ Colibactin and indolimines are small‐molecule metabolites,^[^ ^25^, ^26^ ^]^ whereas CDTs, produced by Gram‐negative bacteria like *Escherichia coli* and *Campylobacter jejuni*, directly cause double‐strand breaks (DSBs) in host DNA, leading to cell cycle arrest or apoptosis.^[^ ^24^, ^27^ ^]^ These CDT toxins exhibit DNase‐like activity, cleaving host DNA and initiating cellular responses aimed at repair or, if irreparable, apoptosis.^[^ ^25^, ^26^, ^27^ ^]^ While numerous studies have elucidated the role of DNase‐type effector proteins in mediating interbacterial competition,^[^ ^28^, ^29^ ^]^ their direct contribution to manipulating host cell functions, particularly in inducing apoptosis, remains largely unexplored. Moreover, the mechanisms by which bacteria utilize secreted effectors to manipulate host apoptosis remain poorly understood.

The cGAS‐STING pathway has emerged as a central player in the host response to DNA damage. The cGAS (cyclic GMP‐AMP synthase) protein detects cytosolic DNA, activating the STING (stimulator of interferon genes) pathway to induce immune responses and apoptosis via downstream effectors like IRF3 and NF‐κB.^[^ ^30^, ^31^ ^]^ Although its role in immunity is well‐documented, the involvement of cGAS‐STING in apoptosis during bacterial infections is a recent discovery and remains understudied.^[^ ^32^, ^33^ ^]^ In particular, how bacteria exploit this pathway to induce apoptosis represents a significant gap in host‐pathogen interaction research.

Although recent studies suggest T6SS may trigger apoptosis,^[^ ^12^ ^]^ the underlying molecular mechanisms remain unclear. In this study, we investigated the DNA‐damage and apoptosis‐inducing functions of T6SS in *Yersinia pseudotuberculosis*, focusing on the trans‐kingdom effector TkeA (T6SS‐secreted trans‐kingdom effector inducing apoptosis). We demonstrated that TkeA, a T6SS‐secreted DNase, is delivered into host cells, enters the nucleus, and induces DNA damage. The resulting fragmented DNA leaks into the cytoplasm, activating cGAS and triggering TNF‐mediated apoptosis. Additionally, T6SS delivers TkeA into target bacterial cells, causing genome degradation and enhancing interbacterial competition. This work provides novel insights into the interplay between bacteria and host cell death pathways, underscoring the innovative dual role of T6SS in microbial competition and host manipulation.

## 2 Results

### 2.1 TkeA is a T6SS‐3 Secreted DNase Effector

Gram‐negative bacteria utilize the type VI secretion system (T6SS) to secrete a variety of effectors that facilitate their survival and replication in complex environments, with DNase enzymes representing a critical effector family.^[^ ^28^, ^29^, ^34^, ^35^ ^]^ To identify new T6SS‐secreted DNases, we conducted a comprehensive search of the *Yersinia pseudotuberculosis* YPIII (*Yptb*) genome, focusing on unidentified valine‐glycine repeat protein G (VgrG) homologs, a structural component of T6SS often associated with adjacent effector genes. The search revealed an orphan VgrG‐containing gene cluster encoding multiple potential T6SS effector‐immunity pairs (*ypk_0764* to *ypk_0773*, Figure S1A, Supporting Information). Among these, YPK_0772 emerged as a candidate nuclease, based on its annotation as a rearrangement hotspot (Rhs) protein containing a GIY‐YIG nuclease domain identified through KEGG SSDB Motif Search (https://www.kegg.jp/ssdb‐bin/ssdb_motif?kid=ypy:YPK_0772).^[^ ^36^ ^]^

**Figure 1:**
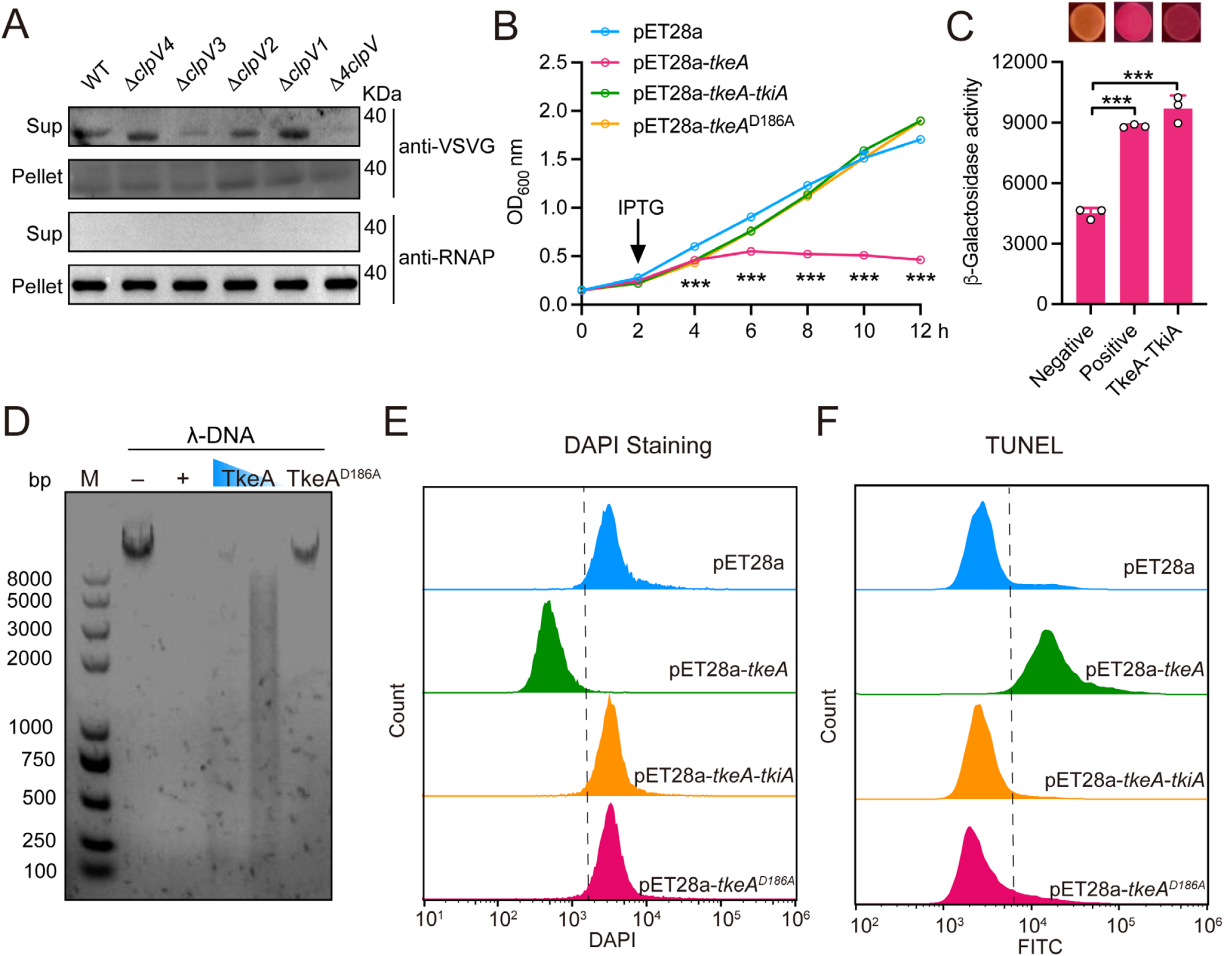
TkeA is a T6SS‐3 secreted DNase effector. A) TkeA is a T6SS3 effector. The indicated strains expressing TkeA‐VSVG were cultured to OD_600_ = 1.6, then total cell pellets (Pellet) and secreted proteins (Sup) in the culture supernatant were isolated and probed for the presence of the TkeA protein with western blotting. Cytosolic RNA polymerase (RNAP) was used as a loading control. The Δ*4clpV* mutant is the mutant strain in which all four essential ATPase genes were deleted. The Δ*clpV1*, Δ*clpV2*, Δ*clpV3* and Δ*clpV4* is the mutant strains that deleted *clpV1*, *clpV2*, *clpV3* and *clpV4*, respectively. B) TkeA is toxic to *E. coli*. Growth curves of *E. coli* BL21(DE3) containing indicated plasmids were determined by measuring OD_600_ from 0 h to 12 h at a 2 h interval. C) Verify the interaction between TkeA and TkiA with the bacterial two‐hybrid assay. Interactions were visualized with the MacConkey maltose plates (upper) and quantified with the β‐galactosidase assay (lower). *n* = 3. D) DNase assays indicating the integrity of λ‐DNA co‐incubated without (−) or with the DNase I control (+, 1 unit per reaction, as per manufacturer's instructions), TkeA (1 or 0.5 µm), or TkeA^D186A^ (1 µm) at 37 °C for 30 min. Reaction products were analyzed using agarose gel electrophoresis. E) Detection of the loss of DNA staining (DAPI) in indicated *E. coli* cells 4 h after IPTG induction. The *X*‐axis corresponds to the 450A filter reading. F) Detection of TkeA‐induced genomic DNA fragmentation after 4 h IPTG induction in the TUNEL assay. DNA fragmentation was detected based on monitoring of fluorescence intensity (indicated on the *X*‐axis) using flow cytometry. The counts resulting from cell sorting are indicated on the *Y*‐axis. *P*  values were calculated using one‐way or two‐way analysis of variance (ANOVA) for multiple comparisons. Error bars represent ± SD. ^***^ *P *< 0.001. See also Figure S1 (Supporting Information).

Secretion assays confirmed that YPK_0772, hereafter referred to as TkeA, is a T6SS‐secreted effector (**Figure** **1**A). Selective inactivation of T6SS‐1 to T6SS‐4 by deleting ATPase ClpV1 to ClpV4 revealed that TkeA secretion is predominantly mediated by T6SS‐3 (Figure 1A). Expression of TkeA in *Escherichia coli* confirmed its toxic activity and such effect was mitigated by co‐expression of the downstream immunity protein TkiA (YPK_0773) (Figure 1B; Figure S1B, Supporting Information). Consistently, the bacterial viability was also reduced by TkeA expression (Figure S1C,D, Supporting Information). The specific interaction between TkeA and TkiA was further validated by a bacterial two‐hybrid assay (Figure 1C). The in vitro DNase assay with purified TkeA confirmed its ability to degrade λ‐DNA, exhibiting a degradation pattern similar to DNase I (Figure 1D). Through random mutagenesis screening, a mutant TkeA protein (TkeA^D186A^) with loss of toxicity in *E. coli* was identified from approximately over 300 candidates (Figure 1B; Figure S1B, Supporting Information). The purified TkeA^D186A^ protein lacked DNase activity (Figure 1D), indicating that the aspartic acid residue at position 186 is critical for its enzymatic function. In vivo DNase activity in *E. coli* of TkeA was further corroborated by DAPI staining and Terminal deoxynucleotidyl transferase dUTP nick‐end labeling (TUNEL) assays (Figure 1E,F; Figure S1D,E, Supporting Information). Additionally, expression of TkeA led to ≈40% of *E. coli* cells undergoing filamentation (Figure S1D,F, Supporting Information), providing additional evidence of DNA damage and halted cell division. These findings showed that TkeA is a DNase effector secreted by T6SS‐3.

### 2.2 TkeA causes DNA Damage in Mammalian Cells

**Figure 2:**
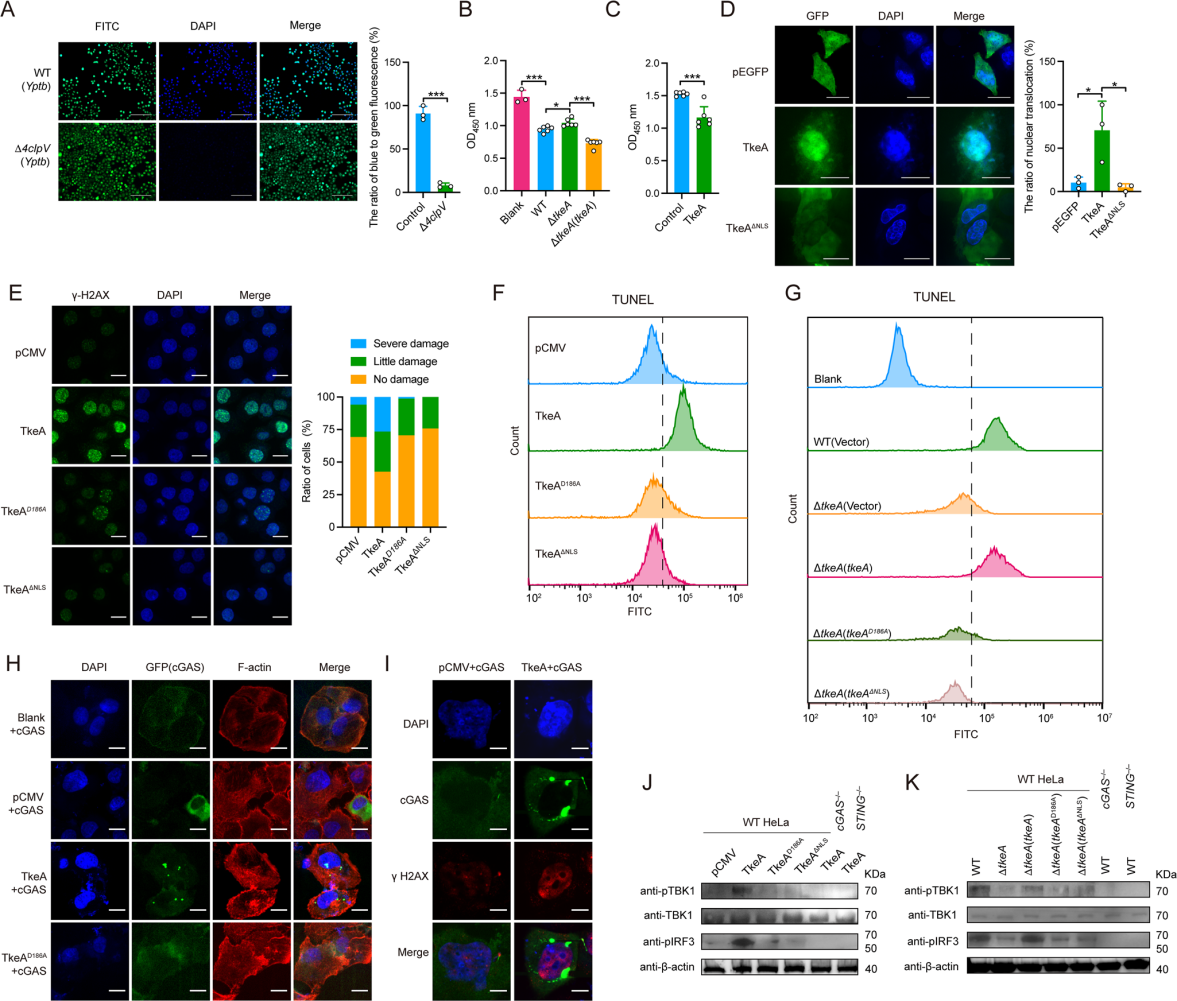
TkeA causes DNA damage in mammalian cells. A) Translocation of TkeA in HeLa cells. HeLa cells were infected with relevant *Yptb* strains introduced with pME6032‐*tkeA*‐*tem* at a MOI of 100 for 1.5 h. Fluorescence microscopy was performed to visualize the translocation of TkeA. Scale bar, 200 µm. The right panel is the quantification of the ratio of blue to green fluorescence. B) The cell counting assay of the non‐infected HeLa cells and those infected with *Yptb* WT, Δ*tkeA*, and Δ*tkeA*(*tkeA*). HeLa cells were incubated with relevant *Yptb* strains at a MOI of 100 for 1.5 h. 10 µL CCK‐8 was added and incubated for 4 h. Absorbance was tested at 450 nm. *n* = 3–6. C) The cell counting assay of the TkeA‐overexpressed HeLa cells. HeLa cells were transfected with pCMV (Control) or pCMV‐*tkeA* vector. After 24 h, 10 µL CCK‐8 was added and incubated for 4 h. Absorbance was tested at OD_450_ nm. *n* = 6. D) Nuclear translocation of TkeA in HeLa cell. pEGFP, pEGFP‐*tkeA*, and pEGFP‐*tkeA* ^Δ^ *^NLS^* mutant plasmids were transfected into HeLa cells for 24 h. Florence microscopy was performed to visualize the translocation of TkeA (green). Nuclear DNA was stained with DAPI (blue). Scale bar, 200 µm. The right panel is the quantification of the ratio of the nuclear translocation. E) Detection of DNA damage led by TkeA in HeLa cell. pCMV, pCMV‐*tkeA*, pCMV‐*tkeA^ΔNLS^* and pCMV‐*tkeA^D186A^* were transfected into HeLa cells for 24 h. Nuclear DNA was stained with DAPI (blue). γ‐H2AX signal was examined using immunofluorescence microscopy (green). The quantification was calculated on the right. Scale bar, 500 µm. F) The indicated HeLa cells transfected with pCMV, pCMV‐*tkeA*, or pCMV‐*tkeA^D186A^* for 24 h by TUNEL‐staining were analyzed by flow cytometry. Data are representative of three independent experiments. G) The indicated HeLa cells infected with WT, Δ*tkeA*, Δ*tkeA* (*tkeA*) and Δ*tkeA* (*tkeA^D186A^*) and Δ*tkeA* (*tkeA^ΔNLS^*) by TUNEL‐staining were analyzed by flow cytometry. Data are representative of three independent experiments. *P*  values were calculated using two‐tailed Student's *t*‐test for paired comparisons or one‐way analysis of variance (ANOVA) for multiple comparisons. H) Translocation of TkeA and activation cGAS. HeLa cells were co‐expressed with TkeA and cGAS‐GFP for 24 h. Fluorescence microscopy was performed to visualize the activation of cGAS. DAPI, nucleus; GFP, cGAS; F‐actin, cytoskeleton. Scale bar, 500 µm. I) Representative immunofluorescence of GFP (cGAS), endogenous γ‐H2AX (DNA damage) in HeLa cells to show the distribution of these proteins. HeLa cells transfected with pCMV, or pCMV‐*tkeA*, and cGAS‐GFP for 24 h. Scale bar, 200 µm. J) Immunoblot analysis of TBK1, phosphorylated TBK1, and IRF3 expression in WT HeLa cells transfected with pCMV, pCMV‐*tkeA*, pCMV‐*tkeA^D186A^*, pCMV‐*tkeA^ΔNLS^* or *cGAS* ^−/−^ and *STING* ^−/−^ HeLa cells transfected with pCMV‐*tkeA* for 24 h. (K) Immunoblot analysis of TBK1, phosphorylated TBK1 and IRF3 expression in WT HeLa cells infected with WT, Δ*tkeA*, Δ*tkeA* (*tkeA*), Δ*tkeA* (*tkeA^D186A^*), Δ*tkeA* (*tkeA^ΔNLS^*) strains and *cGAS* ^−/−^ and *STING* ^−/−^ cells infected with WT strains for 4 h. Error bars represent ± SD. ^*^ *P *< 0.05; ^***^ *P *< 0.001. See also Figures S2 and S3 (Supporting Information).

Since DNA is a common genetic material, it is plausible to hypothesize the effector with DNase activity is a trans‐kingdom effector that targets both prokaryotic and eukaryotic cells. A recent study showed that *Acinetobacter baumannii* uses its T6SS DNase effector TafE to target fungal cells.^[^ ^12^ ^]^ This prompted us to investigate whether TkeA exerts toxic effects in eukaryotic cells. To verify the translocation of TkeA into HeLa cells, we fused the TEM1 (β‐lactamase) reporter protein to the C terminus of TkeA and utilized *Yptb* strains expressing this fusion protein to infect HeLa cells (**Figure** **2**A). The T6SS‐mediated transport of TkeA into HeLa cells was assessed via fluorescence resonance energy transfer (FRET) with the β‐lactamase cleavable substrate CCF2/AM.^[^ ^37^ ^]^ Cells infected with the *Yptb* WT strain expressing TkeA‐TEM1 exhibited blue fluorescence (447 nm), while cells infected with the Δ*4clpV* mutant (lacking all T6SS) predominantly displayed green fluorescence (520 nm), confirming that TkeA was effectively delivered into HeLa cells via T6SS.

The Cell Counting Kit 8 (CCK 8) assay was performed to evaluate the cytotoxicity of TkeA in HeLa and Caco2 cells. Both cells infected with *Yptb* Δ*tkeA* strains showed increased viability compared to those infected with the WT strain or *tkeA* complementation strain, suggesting that TkeA contributes to *Yptb*‐induced cytotoxicity in HeLa and Caco2 cells (Figure 2B; Figure S2A,B, Supporting Information). Additionally, HeLa cells transfected with TkeA‐expressing plasmid exhibited reduced viability compared to cells transfected with either an empty vector, which further confirmed TkeA's toxic effect in mammalian cells (Figure 2C). Of note, the relatively modest cytotoxicity observed in Figure 2B,C, despite TkeA's high DNase activity, may reflect lower intracellular TkeA levels in these assays, host DNA repair mechanisms, or the timing of cytotoxicity measurements. Bioinformatic analysis identified a nuclear localization sequence (NLS: KRKKAHDRKAKK) within TkeA, suggesting that it could localize to the nucleus. This was confirmed by transfecting HeLa cells with GFP‐TkeA. GFP‐TkeA co‐localized with nuclear DAPI staining, whereas a mutant lacking the NLS (GFP‐TkeA^ΔNLS^) was primarily cytosolic (Figure 2D). Interestingly, while other T6SS‐secreted DNases like TepC show some nuclear localization, and Tce1 remains cytosolic (Figure S2C, Supporting Information), only TkeA induces significant DNA damage and apoptosis (Figure S2E, Supporting Information).^[^ ^29^, ^34^ ^]^ These results indicate that TkeA can localize to the nucleus of HeLa cells.

Given that CDT, a known virulence factor with DNase activity can induce DNA damage in mammalian cells,^[^ ^24^ ^]^ we hypothesized that TkeA might also cause DNA damage. We next assessed DNA damage by measuring the phosphorylation of γ‐H2AX, a marker of DNA double‐strand breaks (DSBs).^[^ ^38^ ^]^ HeLa cells expressing TkeA showed elevated levels of phosphorylated γ‐H2AX compared to those transfected with either an empty plasmid or a plasmid harboring the TkeA^D186A^ and TkeA^ΔNLS^ mutant (Figure 2E). Additionally, TkeA expression induced cell cycle arrest, further implicating its role in DNA damage (Figure S2D,E, Supporting Information). Consistently, the HeLa cells infected with the *Yptb* WT and Δ*tkeA*(*tkeA*) strain infection induced higher levels of DNA damage in HeLa cells, whereas the Δ*tkeA* strain, *tkeA^D186A^* complementation strain induced lower levels of DNA damage (Figure S2E, Supporting Information). As the DNase TkeA can lead to DNA fragmentation in prokaryotic cells (Figure 1G), we further measured the presence of DNA termini in TkeA‐transfected HeLa cells. We performed a TUNEL assay in HeLa cells to assess DNA fragmentation. Cells expressing TkeA showed a significant increase in TUNEL‐positive nuclei compared to controls, while cells expressing TkeA^D186A^ or TkeA^ΔNLS^ exhibited few TUNEL‐positive cells (Figure 2F). Similarly, HeLa cells infected with the *Yptb* WT, Δ*tepC*, and Δ*tce1* strain were largely TUNEL‐positive, whereas those infected with the Δ*tkeA* strain showed reduced TUNEL positivity (Figure S2E, Supporting Information). This reduction was reversed by complementation with *tkeA*, but not by the Δ*tkeA* (*tkeA^ΔNLS^*) and *tkeA^D186A^* mutant (Figure 2G). Together, these results demonstrate that the DNase activity of TkeA induces DNA damage in mammalian cells.

### 2.3 TkeA Actives the cGAS‐STING Pathway

DNA damage often leads to DNA fragmentation that leaks into the cytoplasm.^[^ ^39^ ^]^ The cytoplasmic presence of these fragments is known to activate the DNA sensor cGAS, particularly following chromosomal DNA damage.^[^ ^40^ ^]^ To determine whether TkeA activates cGAS, we co‐expressed GFP‐cGAS with either TkeA or the catalytically inactive mutant TkeA^D186A^ in HeLa cells. In cells expressing TkeA, cGAS formed puncta around the nucleus, a pattern not observed in cells expressing TkeA^D186A^ (Figure 2H). This suggests that TkeA‐induced DNA damage leads to the activation of cGAS.

The activation of cGAS typically involves the detection of abnormal DNA in the cytosol or its translocation from the cytoplasm to the nucleus in response to DNA damage.^[^ ^39^, ^41^ ^]^ To investigate the subcellular localization of cGAS, we analyzed the distribution of GFP‐cGAS, DAPI (nuclear genome), and damaged DNA (marked by γ‐H2AX) in HeLa cells. The formation of cGAS‐DNA foci in the cytoplasm of TkeA‐expressing cells confirmed the activation of cytosolic cGAS (Figure 2I). To further validate cGAS activation, we assessed the downstream signaling components of the cGAS‐STING pathway, specifically the phosphorylation of IRF3 and TBK1 in TkeA‐overexpressing HeLa cells. Phosphorylation of IRF3 and TBK1 was strongly induced in WT HeLa cells expressing TkeA, but not in those expressing TkeA^D186A^, TkeA*^ΔNLS^* or the control vector, nor in *cGAS^−/−^* and *STING^−/−^* HeLa cells expressing TkeA (Figure 2J). In addition, *Yptb* WT and Δ*tkeA*(*tkeA*) infection could also induce cGAS activation (Figure S3, Supporting Information). Consistently, WT HeLa cells infected with the *Yptb* WT and Δ*tkeA*(*tkeA*) strain showed elevated levels of pIRF3 and pTBK1, whereas those infected with the Δ*tkeA* strain, *tkeA^D186A^* or *tkeA^ΔNLS^* complementation strain exhibited slightly decreased pIRF3 and pTBK1 levels (Figure 2K). Similarly, no significant induction of pIRF3 and pTBK1 was observed in *cGAS^−/−^* and *STING^−/−^* HeLa cells infected with wild‐type *Yptb* strains. These results confirm that TkeA activates the cGAS‐STING pathway through its DNase activity.

### 2.4 TkeA Elicits Spoptosis in a cGAS‐STING‐Dependent Manner

**Figure 3:**
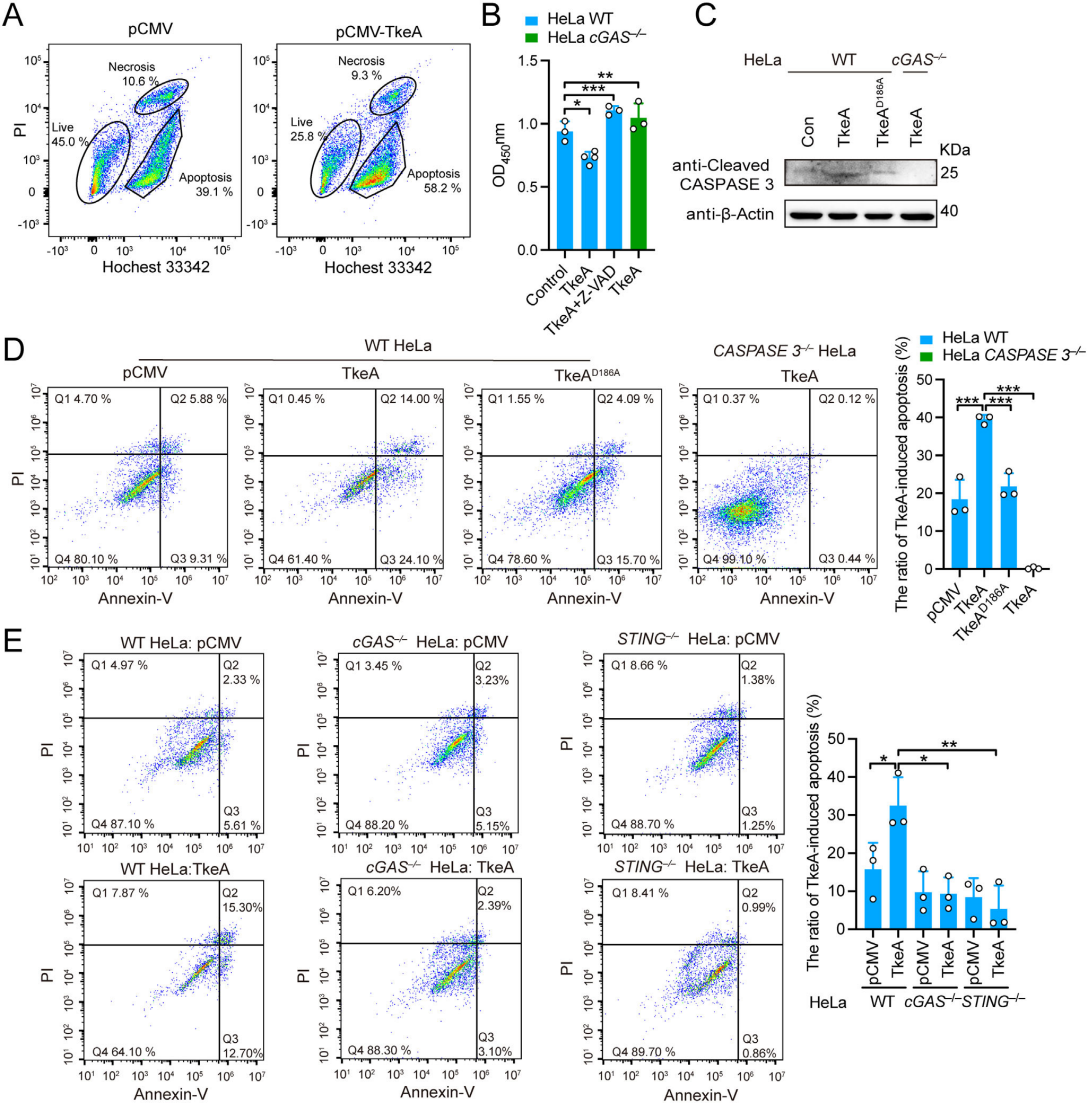
TkeA elicits apoptosis in a cGAS‐STING‐dependent manner. A) HeLa cells transfected with pCMV and pCMV‐*tkeA* for 24 h were collected and stained with Hoechst 33 342/PI. Flow cytometry was used to identify the form of cell death. B) The cell counting assay of the TkeA‐overexpressed WT HeLa cell treated with or without z‐VAD‐FMK and TkeA‐overexpressed *cGAS* ^−/−^ HeLa cells. WT HeLa cells were transfected with pCMV (Control), pCMV‐*tkeA* and pCM,V‐*tkeA* treated with or without z‐VAD‐FMK (30 µm for 6 h). *cGAS* ^−/−^ HeLa cells were transfected with pCMV‐*tkeA*. After 24 h, 10 µL CCK‐8 was added and incubated for 4 h. Absorbance was tested at OD_450_ nm. *n* = 3–4. C) Immunoblot analysis of cleaved CASPASE 3 expression in WT HeLa cells transfected with pCMV, pCMV‐*tkeA*, and pCMV‐*tkeA^D186A^* and *cGAS* ^−/−^ HeLa cells were transfected with pCMV‐*tkeA* for 24 h. D) HeLa cells transfected with pCMV, pCMV‐*tkeA* and pCMV‐*tkeA^D186A^*, and *CASPASE 3* ^−/−^HeLa cells transfected with pCMV‐*tkeA* for 24 h were collected and stained with Annexin V/PI. Flow cytometry was used to identify the cell apoptosis. The right panel is the quantification of the ratio of TkeA‐induced apoptosis. E) WT, *cGAS* ^−/−^ and *STING* ^−/−^ HeLa cells transfected with pCMV or pCMV‐*tkeA* for 24 h and were collected and stained with Annexin V/PI. Flow cytometry was used to identify the cell apoptosis. The right panel is the quantification of the ratio of TkeA‐induced apoptosis. Data in (A), (D), and (E) are from at least three biological replicates. *P*  values were calculated using one‐way analysis of variance (ANOVA) for multiple comparisons. Error bars represent ± SD. ^*^ *P *< 0.05; ^**^ *P *< 0.01; ^***^ *P *< 0.001. See also Figure S4 (Supporting Information).

DNA damage is a well‐known trigger for cell death and programmed cell death (PCD) including apoptosis, pyroptosis, and necroptosis.^[^ ^42^ ^]^ Previous studies have reported that *Yersinia* infection can induce various forms of cell death, including apoptosis and necrosis.^[^ ^43^, ^44^ ^]^ To probe which PCD is elicited by TkeA, we performed Hoechst 33 342/PI double staining. The presence of a lower PI signal indicated that TkeA triggers cell apoptosis but not necrosis (**Figure** **3**A). Furthermore, the pan‐caspase inhibitor z‐VAD‐FMK inhibited TkeA‐induced cell death (Figure 3B), supporting that apoptosis occurs upon TkeA transfection. The introduction of TkeA into HeLa cells also significantly increased the expression of cleaved CASPASE 3, the executioner of apoptosis (Figure 3C). In addition, fluorescence staining further confirmed the activation of CASPASE 3 and apoptosis in TkeA‐expressing HeLa cells (Figure S4A, Supporting Information). These findings provide compelling evidence that TkeA induces apoptosis.

We further confirmed the induction of apoptosis by TkeA by Annexin V‐FITC/PI double staining. Transfection of TkeA resulted in apoptosis in ≈38.1% of cells, compared to 15.19% and 19.79% for pCMV and TkeA^D186A^, respectively (Figure 3D). As expected, the ratio of apoptotic to non‐apoptotic cells in *CASPASE 3^−/−^* HeLa cells transfected with TkeA was significantly lower than in WT HeLa cells. Furthermore, we explored the contribution of TkeA to *Yptb‐*induced apoptosis by using *Yptb* WT, Δ*tkeA*, TkeA complemented strain Δ*tkeA*(*tkeA*) and catalytically inactive TkeA^D186A^ complemented strain Δ*tkeA*(*tkeA^D186A^*) to infect HeLa cells. Consistently, higher levels of apoptosis were observed in the *Yptb* WT and Δ*tkeA*(*tkeA*) infected HeLa cells, compared to those infected with the Δ*tkeA* strain or Δ*tkeA*(*tkeA^D186A^*) strain (Figure S4B, Supporting Information).

It has been reported that the leakage of DNA into the cytoplasm may activate the DNA sensor cGAS.^[^ ^45^ ^]^ To detect the relationship between the cGAS‐STING signaling pathway and TkeA‐induced cell apoptosis, we examined the cell viability in *cGAS^−/−^* HeLa cells expressing TkeA. Compared to the WT HeLa cells expressing TkeA, the cell viability of *cGAS^−/−^* HeLa cells was restored significantly (Figure 3B), indicating that cGAS is involved in TkeA‐induced apoptosis elicited by TkeA. Consistently, TkeA transfection‐induced cleaved CASPASE 3 was markedly downregulated in *cGAS^−/−^* HeLa cells (Figure 3C). Moreover, we performed the Annexin V/propidium iodide staining assay by transfecting TkeA into WT, *cGAS^−/−^* and *STING^−/−^* HeLa cells. Compared with WT HeLa cells, the TkeA‐induced apoptosis was strongly reduced in *cGAS^−/−^* and *STING^−/−^* cells (Figure 3E). Together, these results showed that TkeA elicits apoptosis in a cGAS‐STING‐dependent manner.

### 2.5 The cGAS‐STING‐TNF Signaling Pathway is Implicated in TkeA‐Induced Apoptosis

**Figure 4:**
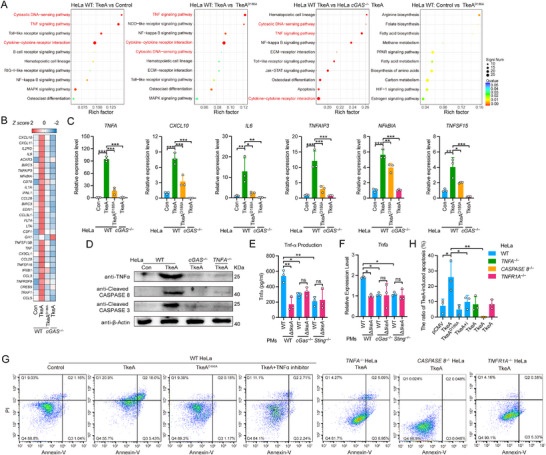
The cGAS‐STING‐TNF signaling pathway is implicated in TkeA‐induced apoptosis. A) Bubble chart of the top 10 significantly KEGG enriched pathways. In bubble charts, the *Y*‐axis represents KEGG pathway terms; the *X*‐axis indicates the Rich factor. The dot dimension corresponds to the number of genes of KEGG terms, and the dot color represents different *P* value ranges. B) Heatmap of RNA‐seq analysis which was made by calculating the RPKM. *n* = 3. C) qRT‐PCR analysis of gene expression in WT HeLa cells transfected with pCMV, pCMV‐*tkeA*, and pCMV‐*tkeA^D186A,^* or cGAS^−/−^HeLa cells transfected with pCMV‐*tkeA*. D) Immunoblot analysis of protein expression in WT, *cGAS* ^−/−^ and *TNFA* ^−/−^ HeLa cells transfected with pCMV and pCMV‐*tkeA*. E) ELISA analysis of TNFα production in C57BL/6 wild‐type, *cGas* ^−/−^ and *Sting* ^−/−^ mouse PMs infected with *Yptb* WT and Δ*tkeA* for 4 h at an MOI of 100. *n * =  3. F) qRT‐PCR analysis of gene expression in C57BL/6 wild‐type, *cGas* ^−/−^ and *Sting* ^−/−^ mouse PMs infected with *Yptb* WT and Δ*tkeA* for 4 h at an MOI of 100. *n*  =  3. G) WT HeLa cells were transfected with pCMV, pCMV‐tkeA, and 10 µm TNFα inhibitor Neochlorogenic acid was added in the pCMV‐tkeA group. *TNFA^−/−^* Cells, *CASPASE 8^−/−^* or *TNFR1A^−/−^* cells were also transfected with pCMV‐tkeA. All cells were collected and stained with Annexin V/PI. Flow cytometry was used to identify the cell apoptosis. H) The quantification of the ratio of TkeA‐induced apoptosis. WT HeLa cells were transfected with pCMV, pCMV‐*tkeA*, and 10 µm TNFα inhibitor Neochlorogenic acid was added in the pCMV‐*tkeA* group. *TNFA* ^−/−^ Cells or *CASPASE 8* ^−/−^ cells were also transfected with pCMV‐*tkeA*. All cells were collected and stained with Annexin V/PI. Flow cytometry was used to identify the cell apoptosis. *n*  =  3. *P*  values were calculated using one‐way or two way analysis of variance (ANOVA) for multiple comparisons. Error bars represent ± SD. ^*^ *P *< 0.05; ^**^ *P *< 0.01; ^***^ *P *< 0.001; ns, not significant. See also Figures S5 and S6 (Supporting Information).

The cGAS‐STING pathway can trigger apoptosis through multiple mechanisms.^[^ ^46^ ^]^ To investigate the mechanisms underlying TkeA‐induced apoptosis downstream of the cGAS‐STING pathway, we performed RNA sequencing (RNA‐seq) analysis on WT HeLa cells expressing pCMV, TkeA, and catalytically inactive TkeA^D186A^, as well as *cGAS^−/−^* HeLa cells expressing TkeA. Principal Component Analysis (PCA) showed distinct clustering for each group (Figure S5A, Supporting Information), and a Venn diagram highlighted the differentially expressed genes across the four groups (Figure S5B, Supporting Information). To identify the signaling pathways involved in cGAS‐STING‐dependent TkeA‐induced apoptosis, we focused on genes differentially expressed in the TkeA‐transfected WT HeLa group compared to other groups. In particular, those genes that downregulated in pCMV, TkeA^D186A^‐transfected WT HeLa group, and TkeA‐transfected *cGAS^−/−^* HeLa group, as compared to the TkeA‐transfected WT HeLa group, were used to perform KEGG pathway enrichment analysis. Another criterion was gene expression that exhibited no significant difference between pCMV‐ and TkeA^D186A^‐transfected WT HeLa cells. The most enriched pathways were the cytosolic DNA‐sensing and TNF signaling pathways (**Figure** **4**A). Notably, many TNF signaling genes were upregulated in TkeA‐transfected WT HeLa cells (Figure 4B).

To validate the RNA‐seq findings, we measured the expression of several TNF signaling pathway genes. TkeA transfection led to significantly higher expression of these TNF‐related genes in WT HeLa cells (Figure 4C), while the expression of *IFNB1* was not affected (Figure S5C, Supporting Information). To confirm that TkeA induces TNF signaling in a cGAS‐STING‐dependent manner, we measured TNFα expression in *cGAS^−/−^* HeLa cells following TkeA transfection. TkeA significantly increased TNFα protein levels in WT HeLa cells, but this phenotype was diminished in *cGAS^−/−^* cells (Figure 4D). We further tested this in HeLa cells infected with *Yptb* WT and Δ*tkeA* strains. *Yptb* WT infection induced higher *TNFA* and *IL1B* mRNA expression compared to Δ*tkeA* infection (Figure S5D, Supporting Information). Previous studies have highlighted the importance of macrophages in mediating immune responses to *Yptb* infection, including the activation of inflammatory pathways and modulation of cell death. Thus, the mouse peritoneal macrophages (PMs) were used to support *Yptb* infection. Similarly, *Yptb* WT infection induced higher TNFα secretion in mouse peritoneal macrophages (PMs), which was attenuated in *cGas* ^−/−^ and *Sting* ^−/−^ PMs (Figure 4E). Of note, the mRNA expression of *Tnfa* was significantly decreased in *cGas* ^−/−^ and *Sting* ^−/−^ PMs (Figure 4F). Consistently, *Yptb* Δ*tkeA* infection led to a decreased *Tnfa* expression and TNFα secretion in PMs compared to WT strain infection (Figure 4E,F). In addition, the *Yptb* WT infection‐elicited apoptosis was also attenuated in *cGas* ^−/−^ and *Sting* ^−/−^ PMs (Figure S5E, Supporting Information). Together, these results demonstrated that the cGAS‐STING pathway is involved in TkeA‐induced TNF production.

CASPASE 8 is a critical component of the TNF signaling pathway, responsible for activating CASPASE 3 and initiating apoptosis.^[^ ^19^ ^]^ To explore whether TkeA‐induced activation of CASPASE 8 and CASPASE 3 is dependent on the TNF signaling pathway, we generated *TNF* ^−/−^ HeLa cells. TkeA transfection significantly increased cleaved CASPASE 8 protein expression in WT HeLa cells, but this was not detected in *cGAS* ^−/−^ HeLa and *TNF* ^−/−^ HeLa cells (Figure 4D). Additionally, using a TNFα inhibitor (neochlorogenic acid), we found that TkeA‐induced apoptosis was markedly reduced. In addition, TkeA‐induced apoptosis was diminished in *TNF* ^−/−^ cells. In addition, *Yptb* WT exhibits a survival advantage over Δ*tkeA* in wild‐type cells, but this advantage is reduced in *cGAS* ^−/−^, *STING* ^−/−^, *TNFA* ^−/−^ and *CASPASE 3* ^−/−^ HeLa cells, suggesting that TkeA‐induced apoptosis enhances bacterial survival (Figure S5F, Supporting Information). Consistently, the deletion of *CASPASE 8* and TNFR1A markedly suppressed TkeA‐induced apoptosis (Figure 4G,H). Collectively, these results demonstrate that TNF signaling is crucial for cGAS‐STING‐dependent TkeA‐induced apoptosis.

### 2.6 TkeA Exerts Anti‐Prokaryotic and Anti‐Eukaryotic Functions in Mice's Gut

**Figure 5:**
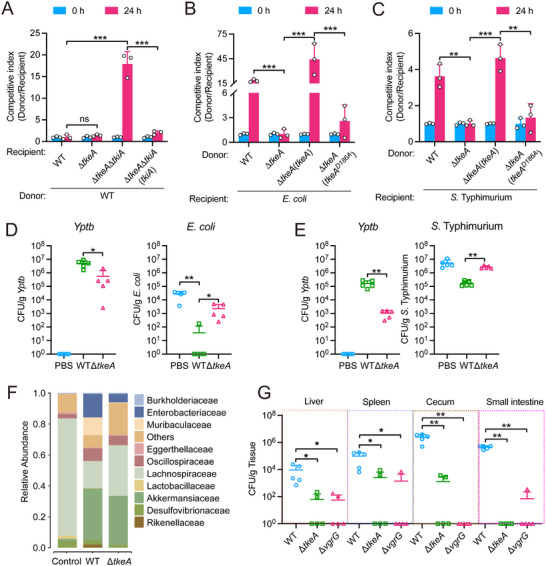
TkeA exerts anti‐prokaryotic functions in mice gut. A) Intra‐species growth competition between the indicated *Yptb* donor and recipient strains at 0 and 24 h. An equal amount of donor and recipient strains were mixed and grown on a solid medium for 24 h at 26 °C. The CFU ratio of donor/recipient strains was calculated based on plate counts. *n* = 3. B,C) TkeA participates in an interference competition. Inter‐species competition between the specified *Yptb* donor strains and recipient bacteria such as *Escherichia coli* (B) and *Salmonella* Typhimurium (C) in M9 solid agar. The donor and recipient strains were combined in equal proportions and thereafter cultured for 24 h at 30 °C. The *Y*‐axis represents the CFU ratio of the donor and recipient strains. D,) Streptomycin‐treated mice were colonized with 5×10^8^ CFUs of *E. coli* (D) or *S*. Typhimurium (E) at day 1, then challenged with 5×10^8^ CFUs of WT *Yptb*, Δ*tkeA* or PBS buffer at day 2. Animals were sacrificed on day 3, and surviving *E. coli* (D, right) or *S*. Typhimurium (E, right) and their corresponding *Yptb* strains (D, left; E, left) in the cecum were counted. PBS was used as the negative control. *n* = 5. F) The analysis of the 16S rRNA gene amplicon of the cecal contents of mice that were orally gavaged with 10^9^ CFUs of different *Yptb* strains. Family‐level distribution of native gut microbiota was shown under three treatments. *n* = 4. G) Mice were orally gavaged with 10^9^ CFUs of different *Yptb* strains. Homogenates of the liver, spleen, cecum, and small intestine were plated to determine the bacterial CFU counts per gram of organs at 24 h post‐infection. n = 4 – 5. *P*  values were calculated using the two‐way analysis of variance (ANOVA) for multiple comparisons. *P*  values in (D), (E), and (G) calculated using the Mann‐Whitney test. Error bars represent ± SD. ^*^ *P *< 0.05; ^**^ *P *< 0.01; ^***^ *P *< 0.001; ns, not significant. See also Figure S7 (Supporting Information).

Successful colonization in the mouse gut requires bacteria to outcompete resident microbiota. To assess the role of TkeA in bacterial antagonism, we performed contact‐dependent growth competition assays. The WT donor strain exhibited a 16‐fold growth advantage over the Δ*tkeA*Δ*tkiA* recipient, which was abolished upon expressing immunity protein TkiA in the recipient strain (**Figure** **5**A). The antagonistic role of TkeA was further supported by interspecies competition assays. Co‐incubation of *Yptb* WT with *E. coli* (Figure 5B) or *Salmonella* Typhimurium (Figure 5C) for 24 h revealed the competitive advantage for *Yptb*, while deletion of *tkeA* significantly reduced this advantage. Complementation with WT TkeA, but not the catalytically inactive TkeA^D186A^, restored this competitive advantage, indicating TkeA's role in *Yptb*’s fitness against *E. coli* and *S*. Typhimurium relies on its DNase activity. To test this in vivo, mice pre‐treated with antibiotics were orally gavaged with *E. coli* (Figure 5D) or *S*. Typhimurium (Figure 5E) on day 1 and infected with the indicated *Yptb* strains on day 2. After 24 h, the intestinal burden of both *E. coli* and *S*. Typhimurium was significantly reduced in mice infected with *Yptb* WT compared to those infected with Δ*tkeA*, indicating that TkeA is crucial for in vivo competition.

To explore TkeA's impact on gut microbiota, we performed 16S rRNA gene sequencing on cecal contents. *Yptb* infection significantly decreased gut microbiota diversity compared to controls, with Δ*tkeA* mutant strains showing a weaker impact on taxonomic diversity than the WT (Figure S6A, Supporting Information). Beta diversity analysis via PCoA revealed substantial segregation in bacterial compositions among the groups (Figure S6B, Supporting Information). *Yptb* WT infection led to marked decreases in the phyla Verrucomicrobiota and Bacillota, with concurrent increases in Bacteroidota and Pseudomonadota (Figure S6C, Supporting Information). At the family level, *Yptb* WT infection resulted in decreased abundances of Eggerthellaceae, Deferribacteraceae, and Lachnospiraceae, while Enterobacteriaceae, Rikenellaceae, Odoribacteraceae, Muribaculaceae, and Akkermansiaceae were increased (Figure 5F).

Many enteric pathogens utilize the T6SS to eliminate symbionts and occupy niches within the host.^[^ ^47^, ^48^ ^]^ To investigate TkeA's role in *Yptb* colonization of mammalian organs, we orally inoculated antibiotic‐pretreated and untreated mice with *Yptb* WT, Δ*tkeA*, and Δ*vgrG* strains. Colonization levels in the liver, spleen, cecum, and small intestine were measured 24 h post‐infection. The result showed that Δ*tkeA* and Δ*vgrG* strains exhibited reduced colonization compared to WT in both antibiotic‐pretreated and untreated mice (Figure 5G; Figure S6D, Supporting Information), indicating that TkeA‐mediated bacterial antagonism may facilitate bacterial colonization by outcompeting gut commensals.

### 2.7 TkeA Induces Apoptosis in Mice's Gut and Contributes to the Virulence of *Yptb*

**Figure 6:**
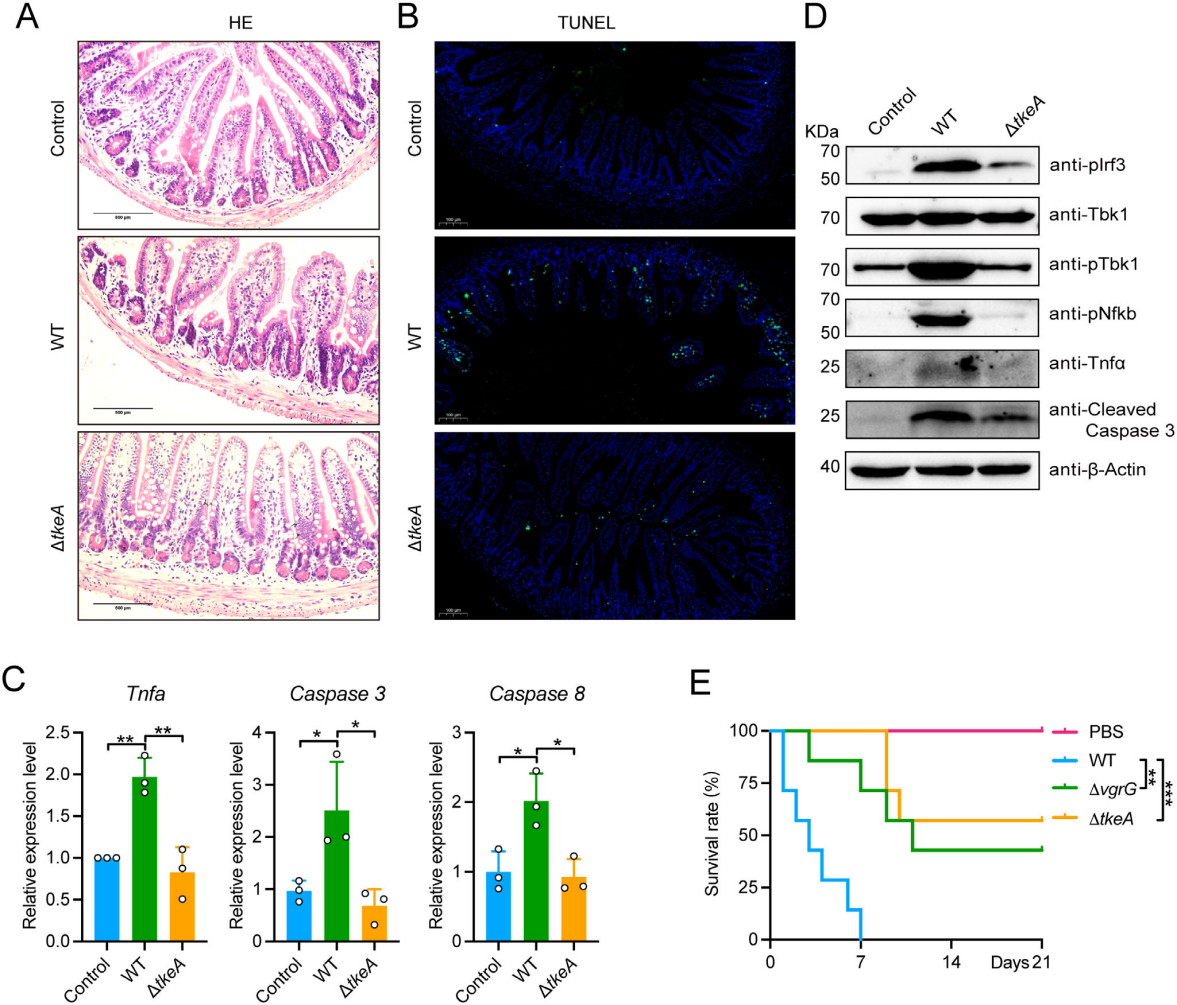
TkeA damages the intestinal epithelial cells of mice. A) Representative microscopic pictures of H & E staining (40 ×, Scale bar, 500 µm) in mice colon infected with PBS, *Yptb* WT or Δ*tkeA* strain. B) Representative microscopic pictures of TUNEL apoptosis assay analysis of cell apoptosis (20 ×, Scale bar, 100 µm) in mice colon infected with PBS, *Yptb* WT or Δ*tkeA* strain. C) qRT‐PCR analysis of gene expression in mice infected with PBS, *Yptb* WT or Δ*tkeA* strain. D) Immunoblot analysis of protein expression in the small intestine from BALB/c mice infected with PBS, WT, and Δ*tkeA* strain. E) Six‐week‐old female BALB/c mice were orally gavaged with indicated *Yptb* strains, which were washed with sterile PBS twice. The survival in different groups of mice was monitored daily for three weeks to establish the survival rate. *n* = 7. *P*  values were calculated using one‐way analysis of variance (ANOVA) for multiple comparisons. *P*  values in E) calculated using the Log‐rank test. Error bars represent ± SD. ^*^ *P *< 0.05; ^**^ *P *< 0.01; ^***^ *P *< 0.001.

To assess TkeA's effect on *Yptb*‐induced toxicity in vivo, we infected mice with PBS, *Yptb* WT, or Δ*tkeA* by oral gavage. Histopathological examination of cecal tissue from mice infected with Yptb WT revealed significant mucosal abscission, disorganized epithelial cell structure, and submucosal expansion (**Figure** **6**A), whereas mice infected with the Δ*tkeA* strain showed no discernible pathological alterations. TUNEL assays further supported these findings, with a markedly higher number of TUNEL‐positive cells in the intestines of WT‐infected mice compared to those in the PBS and Δ*tkeA* groups (Figure 6B). Similarly, TkeA also induced apoptosis in epithelial cells (Figure S2A,B, Supporting Information). Additionally, infection with *Yptb* WT resulted in significantly elevated mRNA levels of *Tnfa*, *Caspase 8*, and *Caspase 3* in mouse intestines, whereas these levels were substantially reduced in the Δ*tkeA*‐infected mice (Figure 6C). Correspondingly, protein analysis demonstrated that cleaved CASPASE 3 levels were higher in WT‐infected mice than in those infected with the Δ*tkeA* strain, confirming that TkeA promotes apoptosis in intestinal cells (Figure 6D). Moreover, the phosphorylation of IRF3 and TBK1 was observed in intestinal cells from WT‐infected mice, but to a lesser extent in Δ*tkeA*‐infected mice. This suggests that the cGAS‐STING pathway is activated during *Yptb* infection in a TkeA‐dependent manner. Finally, both Δ*tkeA*‐infected and T6SS‐defective Δ*vgrG*‐infected mice exhibited significantly lower mortality rates compared to those infected with the WT strain (Figure 6E). These findings demonstrate that TkeA acts as a trans‐kingdom effector targeting both bacterial and mammalian cells, facilitating competition against gut bacteria and inflicting damage on intestinal epithelial cells, thus contributing to the overall virulence of *Yptb* in a mouse infection model.

## 3 Conclusion

The T6SS has long been viewed as a potent weapon in the arsenal of Gram‐negative bacteria for its ability to manipulate host cellular processes and mediate interbacterial competition. Our findings reveal a more sinister role: transforming this molecular syringe into a direct trigger of apoptosis in mammalian cells. T6SS was identified for its role in manipulating host cellular responses and the following studies revealed its role in mediating inter‐bacterial competition and promoting survival in diverse ecological niches.^[^ ^49^ ^]^ However, our study reveals a previously unrecognized function of the T6SS, which is capable of directly inducing apoptosis in mammalian cells. This discovery is centered on a trans‐kingdom DNase effector, TkeA, secreted by *Yptb*, which not only outcompete bacterial competitors by degrading their DNA but also inflicts DNA damage in host cells, triggering apoptosis through the cGAS‐STING‐TNF signaling pathway.

In contrast to the previously identified trans‐kingdom DNaseTafE from *Acinetobacter baumannii*, which specifically targets fungal cells and localizes within the cytoplasm of HeLa cells,^[^ ^12^ ^]^ TkeA exhibits the unique capability to enter the nucleus of mammalian cells. Our observations demonstrate that GFP‐labeled TkeA accumulates in the nuclei of HeLa cells, a process notably hindered when its nuclear localization signal is deleted (Figure 2H). This nuclear targeting by TkeA is linked to significant degradation of the host genome, support its role in DNA damage and apoptosis induction. Furthermore, while the impact of TafE on in vivo virulence remains unclear, our study shows that TkeA is important for the virulence of *Yptb* in mouse infection models (Figure 6E). Together, these findings showed that TkeA is a trans‐kingdom effector that could disrupt the nuclei of mammalian cells to induce apoptosis.

Apoptosis is generally seen as a non‐inflammatory form of programmed cell death (PCD) that cells use to prevent excessive inflammatory responses.^[^ ^22^ ^]^ Unlike necrosis, which is a pro‐inflammatory cell death that potentially alerts the immune system,^[^ ^50^ ^]^ apoptosis allows bacteria to evade detection in a manner that is less likely to trigger a strong immune response. While the roles of bacterial secretion systems like T2SS, T3SS, and T4SS in inducing apoptosis have been well‐studied,^[^ ^51^, ^52^, ^53^ ^]^ the function of the T6SS in this context is not as clearly understood. Some studies suggest that T6SS can also induce apoptosis,^[^ ^54^, ^55^ ^]^ but the underlying mechanisms remain unclear. In this study, we showed that the T6SS effector TkeA is translocated into the nucleus of mammalian cells, where its DNase activity causes DNA damage. This damage results in the release of DNA fragments into the cytoplasm, which activates the cGAS‐STING pathway. The activation of this pathway then triggers the TNF signaling cascade, leading to apoptosis through the activation of CASPASE 8. Our findings reveal a novel mechanism by which TkeA induces apoptosis, involving the cGAS‐STING‐TNF signaling pathway.

This mechanism is fundamentally different from how T3SS and T4SS effectors induce apoptosis. T3SS and T4SS often manipulate host signaling pathways or interfere directly with cellular machinery. For instance, T3SS effectors such as YopJ from *Yersinia* inhibit MAPK and NF‐κB pathways, leading to apoptosis through the intrinsic mitochondrial pathway.^[^ ^56^ ^]^ Similarly, *Salmonella enterica* secretes the effector protein SipB, which interacts with CASPASE‐1 to cause pyroptotic cell death in macrophages.^[^ ^57^ ^]^ Another example is the T3SS effector IpaB from *Shigella flexneri*, which activates CASPASE‐1 to induce apoptosis in infected macrophages.^[^ ^58^ ^]^ In contrast, T6SS effectors like TkeA directly damage the host cell's nuclear DNA, activating the cGAS‐STING pathway to trigger apoptosis. Notably, the apoptotic pathway triggered by TkeA is distinct from the intrinsic apoptosis pathway activated by other bacterial toxins, such as the cytolethal distending toxin (CDT). CDT primarily activates the intrinsic mitochondrial pathway through DNA damage‐induced activation of p53.^[^ ^24^ ^]^ For example, CDT from *Aggregatibacter actinomycetemcomitans* can increase the levels of pro‐apoptotic proteins Bid, Bax, and Bak in a p21CIP1/WAF1‐dependent manner in Jurkat^p21−^ cells.^[^ ^59^ ^]^ Our findings present a new mechanism by which secretion system effectors induce apoptosis in host cells via the cGAS‐STING pathway. This expands our understanding of how T6SS effectors function in the complex interactions between bacteria and host cells.

While the cGAS‐STING pathway is essential for activating innate immune defenses, bacteria have evolved T6SS effectors to modulate this pathway. For instance, *Burkholderia pseudomallei* T6SS5‐mediated host cell fusion triggers the cGAS‐STING pathway and induces cell autophagy.^[^ ^60^ ^]^ In our study, we demonstrate that the T6SS effector TkeA damages nuclear DNA in host cells, leading to cGAS‐STING pathway activation and subsequent apoptosis. A key aspect of TkeA in culminating apoptosis is the translocation of fragmented nuclear DNA into the cytoplasm, which then activates the cGAS pathway. Unlike the CDT, which induces apoptosis via the intrinsic pathway,^[^ ^24^ ^]^ TkeA induces cell death through the extrinsic pathway, primarily by activating the cGAS sensor. However, the role of the cGAS‐STING pathway in *Yptb* infection is complex and paradoxical. Our previous study showed that *Yptb* employs a T6SS effector, TssS, that can chelate Mn^2+^ to counteract the STING‐mediated innate immune response.^[^ ^61^ ^]^ This raises important questions about the contribution of the cGAS‐STING pathway to *Yptb* infection progression. We speculate that the HeLa cells used in our study exhibited a minimal type I IFN response to bacterial infection, which dwarfs the *Yptb*‐induced innate immune response and the induction of apoptosis becomes more prominent. The crosstalk between these pathways highlights the complexity of T6SS‐induced cellular responses, where effectors can directly target host cell molecules or modulate intracellular signaling to regulate those responses.

Recent research has shown that damaged nuclear and mitochondrial DNA can induce apoptosis via the cGAS‐STING pathway by activating transcription factors like IRF3 and NF‐κB in HaCaT cells.^[^ ^39^ ^]^ Based on these findings, our study showed that TkeA‐induced apoptosis is mediated through the cGAS‐STING‐TNF pathway. Notably, we found that the pro‐apoptotic effects of TkeA are driven by TNFα activation, which leads to apoptosis. This conclusion is supported by several lines of evidence: 1) transcriptomic analysis revealed an enrichment of cytosolic DNA‐sensing and TNF signaling pathways in TkeA‐expressing HeLa cells; 2) higher TNFα production was observed in *Yptb* WT‐infected cells compared to Δ*tkeA*‐infected cells, with no difference in *cGas^−/−^* and *Sting^−/−^* PMs. Significantly lower TNFα levels were detected in the supernatant of *cGas^−/−^* and *Sting^−/−^* PMs, underscoring the critical role of the cGAS‐STING pathway in mediating TNFα production; 3) inhibition of TNFα function in HeLa cells led to a reduction in apoptosis; and 4) transfection of pCMV‐*tkeA* significantly affected *Tnfa* expression but had little impact on *Ifnb1* expression, indicating that TkeA induces apoptosis through TNFα rather than IFN. These results convincingly demonstrate that TNFα is the key factor mediating DNA damage‐induced apoptosis. Although TNFα is primarily known as a central cytokine in inflammatory reactions, it has also been reported to induce cell death,^[^ ^18^ ^]^ which is not the default response of cells to TNFα. This raises the question of why TNFα preferentially induces apoptosis during *Yptb* infection. One possibility is that the TNF‐TNFR1 cascades active cell death only when one of the cell death checkpoints is inactivated and those checkpoints might be inactivated during *Yptb* infection.^[^ ^18^, ^62^ ^]^ However, further experiments are needed to confirm this.

Chronic activation of STING has been implicated in various inflammatory conditions, including autoimmune diseases and cancer.^[^ ^63^ ^]^ It is possible that DNA damage and STING activation caused by TkeA could potentially contribute to disease progression and increase the risk of carcinogenesis. However, this hypothesis requires further investigation, particularly in the context of gut‐associated bacteria and colorectal cancer (CRC). Interestingly, our study showed that WT *Yptb* strain infection in mice gut caused significant increases in the abundance of microbial families such as Enterobacteriaceae, Akkermansiaceae, and Rikenellaceae, all of which are associated with an increased risk of CRC.^[^ ^64^, ^65^, ^66^, ^67^ ^]^ Conversely, *Yptb* WT strains significantly decreased the abundances of Eggerthellaceae, Lachnospiraceae, and Lactobacillaceae, all of which are associated with a reduced risk of CRC.^[^ ^68^, ^69^ ^]^ This suggests that TkeA‐mediated host DNA damage, combined with alterations in gut microbiota composition, may contribute to *Yptb*‐mediated intestinal carcinogenesis. However, further research is necessary to validate this hypothesis.

In conclusion, the identification of TkeA as a T6SS DNase effector that induces apoptosis through the cGAS‐STING‐TNF axis underscores the complexity and versatility of T6SS in bacterial pathogenesis. This study not only highlights a unique strategy employed by bacteria to manipulate host cell fate but also opens up new avenues for exploring the therapeutic potential of targeting T6SS‐mediated host‐pathogen interactions.

## 4 Experimental Section

### Mouse Studies

Six‐week‐old female mice (BALB/c) were purchased from Beijing Vital River Laboratory Animal Technology Co., Ltd from China. The mouse experimental procedures complied with the Regulations for the Administration of Affairs Concerning Experimental Animals, which were approved by the State Council of the People's Republic of China. The protocol was approved by the Animal Welfare and Research Ethics Committee of Northwest A&F University (Protocol number: XN2023‐1004). The mice were housed in a controlled environment with a temperature of 24 ± 2 °C and a light cycle of 12 h of light followed by 12 h of darkness. They were provided with ad libitum access to food and water.

### Cell Culture

HeLa cells were grown in DMEM media supplemented with 10% heat‐inactivated FBS, 100 U mL^−1^ penicillin, and 100 g mL^−1^ streptomycin at 37 °C with a CO_2_ concentration of 5%. Mouse peritoneal macrophages (PMs) were harvested from mice after intraperitoneal injection with beef extract peptone medium (0.3% beef extract, 1% peptone, 0.5% NaCl, and 6% Soluble starch) for 3 days, and were cultured in RPMI 1640 medium, supplemented with 10% FBS, penicillin (100 U mL^−1^), streptomycin (100 µg mL^−1^), 10 µm sodium pyruvate, 0.1 mM non‐essential amino acids, 50 mM 2‐mercaptoethanol and 25 mm HEPES for 1 day.^[^ ^70^ ^]^

### Bacterial Strains and Growth Conditions

Bacterial strains and plasmids used in this study are listed in Table S1 (Supporting Information). The *Yptb* YPIII strains were cultured in Yersinia‐Luria‐Bertani (YLB) broth (1% tryptone, 0.5% yeast extract, 0.5% NaCl) or M9 minimal medium (Na_2_HPO_4_, 6 g L^−1^; KH_2_PO_4_, 3 g L^−1^; NaCl, 0.5 g L^−1^; NH_4_Cl, 1 g L^−1^; MgSO_4_, 1 mm; CaCl_2_, 0.1 mm; glucose 0.2%, pH 7.0) at 26 °C. *Escherichia coli* and *Salmonella* Typhimurium strains were grown in Luria Bertani (LB) with appropriate antibiotics at 37 °C. The concentrations of antibiotics used in this study were as follows: nalidixic acid, 20 µg mL^−1^; kanamycin, 50 µg mL^−1^; ampicillin, 100 µg mL^−1^; chloramphenicol, 20 µg mL^−1^; tetracycline, 10 µg mL^−1^; streptomycin, 100 µg mL^−1^.

### Plasmid Construction

The primers used in this study are enumerated in Table S2 (Supporting Information). To acquire the expression plasmids, the genes encoding *Yptb* TkeA were amplified using polymerase chain reaction (PCR). Plasmid derivatives were created by digesting the DNA fragment and cloning it into vector pET28a with the same double restriction site. The expression clones of TkiA (YPK_0773) and TkeA‐TkiA were achieved in the same manner. Overlap PCR was applied to create the plasmid pDM4‐Δ*tkeA*, which was then used to create the Δ*tkeA* in‐frame deletion mutant. In summary, the amplification of the upstream fragment and downstream fragment of the *tkeA* gene was carried out using the primer pairs *tkeA*‐M1F‐*Bam*HI/*tkeA*‐M1R and *tkeA*‐M2F/*tkeA*‐M2R‐*Sal*I. Then, both of the PCR fragments were combined using the primer pair *tkeA*‐M1F‐*Bam*HI/*tkeA*‐M2R‐*Sal*I through the process of overlap PCR. The PCR products were digested with *Bam*HI and *Sal*I, and inserted into similar digested suicide plasmid pDM4 to produce pDM4‐Δ*tkeA*. The knock‐out plasmid pDM4‐Δ*tkeA*Δ*tkiA*, and pDM4‐Δ*vgrG* were constructed with the same method.

To construct plasmids used in bacterial two‐hybrid assays, the *tkeA* gene were amplified by PCR using the primer pair *tkeA*‐F‐*Xba*I and *tkeA*‐R‐*Eco*RI. Amplified DNA fragments were digested with restriction enzymes *Xba*I and *Eco*RI, and cloned into the corresponding sites of pKT25 vector. The cloning vectors pUT18C‐*tkiA* were obtained with the same manner. To complement the Δ*tkeA* mutant, primers *tkeA*‐F‐*Spe*I and *tkeA*‐R‐*Sal*I were employed to amplify the *tkeA* gene fragment from *Yptb* genomic DNA. The PCR product was digested with *Spe*I/*Sal*I and ligated into similarly digested pKT100 to produce pKT100‐*tkeA*. The complementary plasmids pKT100‐*tkiA*, pKT100‐*vgrG*, pEGFP‐*tkeA* and pCMV‐*tkeA* were constructed similarly. Plasmid pME6032‐*tkeA*‐vsvg was constructed for protein secretion assay. Briefly, primers *tkeA*‐F‐*Eco*RI and *tkeA*‐R‐*vsvg*‐*Bgl*II were employed to amplify the *tkeA* gene from *Yptb* genomic DNA. The PCR product was digested with *Eco*RI/*Bgl*II and inserted into similarly digested pME6032 to generate pME6032‐*tkeA*‐*vsvg*. To construct TEM1 translocation reporter vector, primers *tkeA*‐F‐*Eco*RI/*tkeA*‐R‐*Bgl*II were utilized to amplify the gene *tkeA* from the *Yptb* genome DNA. Then, the fragment *tkeA* was inserted into the pME6032‐*tem1* with the same digested sites to produce pME6032‐*tkeA*‐*tem1*. The primer pairs *tkeA^D186A^*‐F and *tkeA^D186A^*‐R were used to amplify the complete plasmid pET28a‐*tkeA^D186A^*, pKT100‐*tkeA^D186A^*, pEGFP‐*tkeA^D186A^* and pCMV‐*tkeA^D186A^* using QuickMutation Site‐Directed Mutagenesis Kit. To obtain pCMV‐*cGAS*‐*GFP*, primers *cgas*‐F‐*Hin*dIII/*cgas*‐R‐*Bgl*II were used to amplify the fragment *cGAS* of human. Then, *cGAS* were inserted into pCMV‐C‐EGFP with the same digested sites *Hin*dIII/*Bgl*II. The integrity of the insert in all constructs was confirmed by DNA sequencing.

### Overexpression and Purification of Recombinant Proteins

In order to achieve the expression and purification of recombinant proteins tagged with His_6_ and GST, the plasmid pET28a derivatives were introduced into *Escherichia coli* strains BL21(DE3). The bacteria were cultured in 5 mL of Luria‐Bertani (LB) medium at 37 °C until reached the stationary phase. Then bacteria were re‐inoculated into fresh LB medium at a ratio of 1:100 and cultivated at 37 °C until the optical density at 600 nm (OD_600_) reached a value of 0.40. Subsequently, a concentration of 0.2‐0.5 mm IPTG was added into the growth medium, and the cultivation process was extended for an additional 12 h at 16 °C with 150 rpm. The cells were harvested and subjected to sonication for disruption. Purification was carried out using either the His•Bind Ni‐NTA resin (Novagen, Madison, WI), following the instructions provided by the manufacturer. The purified proteins underwent dialysis against phosphate‐buffered saline (PBS) at 4 °C overnight. All reagents are listed in Table S3 (Supporting Information).

### Protein Secretion Assay

To conduct the secretion assay, *Yptb* strains were cultivated in 3 mL of YLB medium at 26 °C. Subsequently, the cultures were transfected into 300 mL of YLB medium supplemented with 1 mm IPTG until OD_600_ reached 1.60. A total volume of 2 mL of culture solution was obtained, and cell pellets were resuspended in the SDS‐PAGE sample loading buffer. A volume of 280 mL of culture medium was centrifuged at 5,000 rpm for 20 min. Next, the supernatant was centrifuged at a speed of 9,900 rpm for another 50 min. The final supernatant was collected and filtered with a 0.22 µm pore size filter (Millipore, MA). The proteins were collected by filtration with a nitrocellulose filter three times (BA85, Whatman, Germany). The filter was dissolved in 100 µL of SDS loading buffer and incubated at 65 °C for 15 min, then boiled for 10 min to recover the protein present. The protein samples from the total cell pellet and culture supernatant were separated using SDS‐PAGE and subsequently analyzed through the Western blot.

### Bacterial Two‐Hybrid Assay

The bacterial two‐hybrid complementation assays were conducted following the methods outlined in previous studies. In this study, the pKT25 and pUT18C derivatives were co‐transformed into *E. coli* BTH101. The transformed cells were then cultured on a MacConkey plate supplemented with Ampicillin (100 µg mL^−1^), Kanamycin (50 µg mL^−1^), and IPTG (1 mm) at 30 °C. Simultaneously, the plasmid pKT25‐zip/pUT18C‐zip and pKT25/pUT18C were introduced into *E. coli* BTH101, with the former serving as the positive control and the latter as the negative control. The interactions were assessed by employing the MacConkey medium, whereby the presence of a red colony color indicates a protein interaction. The quantification of protein interactions was conducted by measuring the activities of β‐galactosidase in liquid cultures. In summary, overnight cultures were diluted to a concentration of 1% and subsequently cultivated in LB broth supplemented with antibiotics at 30 °C until the OD_600_ reached 1.0. The enzymatic activity of β‐galactosidase was then evaluated using o‐nitrophenyl‐β‐D‐galactopyranoside (ONPG) as the substrate.

### Growth Inhibition Assay

*E. coli* BL21(DE3) strains containing pET28a, pET28a‐*tkeA*, pET28a‐*tkeA^D186A^*, and pET28a‐*tkeA*‐*tkiA* plasmids were cultured in an LB medium. The cultures that had been incubated overnight were standardized to achieve the same optical density and subsequently diluted by a factor of 100 into LB broth supplemented with suitable antibiotics. Following incubation at 26 °C and 180 rpm for 2 h, the expression of recombinant proteins was added with 0.5 mm IPTG. Subsequently, the incubation was sustained under the same condition. The monitoring of cultural growth was conducted by measuring OD_600_ at regular intervals of 2 h.

### Western Blot Analysis

The protein samples underwent resolution through SDS‐PAGE and subsequent transfer onto PVDF membranes (Millipore, MA). Subsequently, the membrane was blocked using a 5% (w/v) BSA solution for 8 h at 4 °C. Following this, the membrane was incubated with primary antibodies overnight at 4 °C. The antibodies used in this study were as follows: anti‐VSVG at a dilution of 1:1000; anti‐RNAP at a dilution of 1:400; and anti‐His at a dilution of 1:500. The rest of the antibodies were used at a dilution of 1:1000. The membrane was washed in TBST buffer, consisting of 50 mm Tris, 150 mm NaCl, 0.05% Tween 20, and pH 7.4. Subsequently, it was incubated with secondary antibodies for 4 h at 4 °C. Following the incubation, the membrane was washed 5 times using TBST buffer. The signals were detected by employing the ECL plus kit in conjunction with a Chemiluminescence imager (Tanon 5200Multi, Beijing).

### Construction of Mutant Library by epPCR

The plasmid pET28a‐*tkeA* was subjected to error‐prone PCR (epPCR) using the QuickMutation Random Mutagenesis Kit. The primers *tkeA*‐F‐*Eco*RI and *tkeA*‐R‐*Sal*I were utilized according to the manufacturer's instructions. The experimental polymerase chain reaction (epPCR) program was implemented in the following process: 94 °C for 3 min, 30 cycles of 30 s at 94 °C, 30 s at 55 °C, and 30 s at 72 °C, followed by 10 min at 72 °C final extension. The amplified DNA fragments obtained from PCR were subjected to gel purification. Subsequently, these purified fragments were digested using *Eco*RI and *Sal*I. The resulting digested fragments were then inserted into pET28a plasmids that had been similarly treated with *Eco*RI and *Sal*I enzymes. The ligation mixture was transformed into BL21(DE3). Transformants lost toxicity were screened in an LB medium containing 0.3 mm IPTG and were further verified by cloning the mutated alleles of *tkeA* into a new vector. The mutations were identified through the process of DNA sequencing analysis.

### DNase Assay

The TkeA protein purified was incubated with λ DNA in a reaction buffer consisting of 20 mm MES, 100 mm NaCl, 2 mm MgCl_2_, and pH 6.9. In all, 4 mm EDTA was supplemented in the reaction system. The DNA hydrolysis was conducted at 37 °C for 30 min. The state of DNA integrity was subsequently assessed using 0.7% agarose gel electrophoresis.^[^ ^29^, ^34^ ^]^

### DAPI Staining and Flow Cytometry Analysis for Bacteria

The DAPI staining and flow cytometry analysis were conducted according to the methods described before. The *E. coli* BL21(DE3) strain, harboring the pET28a plasmid or its derivatives expressing TkeA alone (pET28a‐*tkeA*) or TkeA‐TkiA together (pET28a‐*tkeA*‐*tkiA*), was cultured overnight. Subsequently, the culture was diluted with the ratio of 1/100 into LB broth and incubated at 37 °C with 180 rpm. After 2 h, the samples were stimulated through the introduction of 0.5 mm IPTG. Subsequently, cultivation was sustained for an additional 4 h at 26 °C. The collected cells were washed by phosphate‐buffered saline (PBS). The cells were fixed and incubated in a solution containing 0.3% Triton X‐100 in PBS for 5 min. Following this, the cells were stained using a concentration of 10 µg mL^−1^ of DAPI for 5 min at 37 °C. The stained cells were then washed with PBS three times. Finally, the cells were examined using either a fluorescence microscope (Andor Revolution‐XD, Britain) or flow cytometry (Beckman, CytoFLEX). A total of 10000 cells were collected for each sample and analyzed using FlowJo_V10 software.

### Quantitative Real‐Time PCR (qRT‐PCR)

Exponentially growing strains were subjected to total RNA isolation using the RNAprep Pure Cell/Bacteria Kit in conjunction with the DNase I Kit. The concentration of RNA was determined by the NanoDrop 2000 spectrophotometer (Thermo Fisher Scientific, USA). The measurement of mRNA abundance in each of the samples was conducted by the TransStart Green qPCR Super‐Mix and the Bio‐Rad CFX96 Real‐Time PCR Detection System (Bio‐Rad, USA), following the instructions provided by the manufacturers. The primers used in this investigation are listed in Table S2 (Supporting Information). To standardize the results, the internal standard of relative abundance of 16S rRNA was employed.

### Intra‐Species and Inter‐Species Competition In Vitro

The intra‐species competition assays were conducted following the method described with slight modification. In summary, strains grown overnight were washed and adjusted to an optical density of 1.0 at OD_600_ using an M9 medium. These adjusted strains were then combined to conduct a competition. The donor‐to‐recipient ratio at the beginning of the experiment was 1:1. The co‐cultures were then introduced into LB/M9 medium and incubated at 30 °C for 24 h. To perform inter‐species competition assays, *Yptb* strains and *Escherichia coli* (DH5α) or *Salmonella* Typhimurium strains were cultured overnight. The resulting cultures were washed three times using M9 liquid and subsequently adjusted to an optical density of 1.0 at OD_600_. The *Yptb* strains and target strains were combined in equal proportions and subjected to incubation at 30 °C for 24 h. The CFU ratio of the donor and recipient strains was assessed through plate counts at corresponding time intervals subsequent to the competition. The data obtained from all competitions were analyzed using the one‐way or two‐way ANOVA test. The presented results represent the average of a single representative assay that was conducted three times.

### Mouse Infection

Mid‐exponential phase *Yptb* strains were grown in YLB medium at 26 °C, and washed with saline water twice. Six‐week‐old female mice were orally gavaged with 100 µL indicated *Yptb* strains with 10^9^ CFU, and the survival rate of the mice was measured daily for 21 days.^[^ ^29^, ^34^, ^61^ ^]^ For histopathological analysis, three mice per group (PBS, *Yptb* WT, Δ*tkeA*) were assessed. For each mouse, three sections from the colon were prepared and analyzed by H&E staining and TUNEL assay.

### Murine Colonization Assay

Six‐week‐old female BALB/c mice were adapted in the laboratory for three days. Subsequently, they were orally gavaged with 10^9^ CFUs of the corresponding *Yptb* strains. The mice were then observed and monitored for either 24 or 48 h. At the end of the experiment, mice were sacrificed, and the liver, spleen, cecum, and small intestine tissue were ground and plated on selective YLB antibiotic plates for CFU enumeration.

### Translocation Assay for TkeA::TEM1 Fusions

The translocation experiment was carried out as previously described. TEM1 fusion TkeA expressing bacterial strains were incubated with HeLa cells (at a MOI of 100) for 1.5 h in 96‐well black‐wall, clear‐bottom plates. After three washes in PBS, HeLa cells were treated for 90 min at room temperature with CCF2‐AM (LiveBLAzer FRET‐B/G Loading Kit). Fluorescence was measured using a microtiter plate reader at an excitation wavelength of 410 nm following the manufacturer's instructions. Translocation was shown using a comparison of the cleaved (blue, 450 nm) and uncleaved (green, 520 nm) signals. A Nikon fluorescent microscope (Nikon, Japan) was used to look at the materials up close and personal.

### CCK‐8 Assay

Cell toxicity experiments were conducted following the guidelines provided by the manufacturer of Cell Counting Kit 8 (CCK8). HeLa cells were inoculated into 96‐well plates and then exposed to the specified *Yptb* strains (MOI = 100) or transfected with pCMV, TkeA, or TkeA^D186A^ constructs for 24 h. The cells were stained using a regnant solution in the kit with a concentration of 10% (v/v). After 30 min, the absorbance at 450 nm was quantified using a microplate reader.

### Fluorescence Assay and Immunofluorescence Assay

Cells were seeded on Glass Bottom Cell Culture Dish (Biosharp) and were transfected with the indicated plasmid for 24 h. Treated cells were washed with PBS buffer twice and fixed with Immunol Staining Fix Solution for 15 min at room temperature. The fixative was removed, and the cells were washed three times with PBS Buffer for 3–5 min each wash. As for the fluorescence assay, the cells were incubated with PBS containing 0.3% Triton X‐100 for 5 min, and stained using TRITC Phalloidin. The nuclear stain (DAPI) was added for 5 min and cells were washed 3 times. As for the Immunofluorescence assay, the DNA Damage Assay Kit by γ‐H2AX Immunofluorescence was used to do the following steps. Briefly, after blocking the cells for an hour, γ‐H2AX Rabbit mAb was added to incubate for 1 h at room temperature. Cells were washed 3 times with PBS for 5–10 min each time. Next, after incubating with anti‐rabbit 488 or anti‐rabbit 555 for 1 h, the cells were washed twice with PBS for 5–10 min each time. DAPI was added for 5 min and cells were washed 3 times. γ‐H2AX staining exhibits green or red fluorescence and DAPI staining of nuclei exhibits blue fluorescence. Images were acquired via a high‐speed rotary disc‐type fluorescence confocal microscope (Andor Revolution‐XD, UK). For quantification of DNA damage, the γ‐H2AX immunofluorescence assay was performed. Cells were categorized based on the extent of γ‐H2AX fluorescence: samples in which more than 50% of cells exhibited strong green fluorescence were classified as having severe DNA damage, whereas those with fewer than 50% γ‐H2AX‐positive cells were considered to have slight damage. At least three images per group were analyzed, each containing more than 50 cells. The number of γ‐H2AX foci per cell was quantified using ImageJ software (version 1.53). The TUNEL assay was employed to detect intracellular DNA damage. Upon genomic DNA fragmentation, exposed 3′‐OH termini were labeled with a green fluorescent probe (FITC) in a reaction catalyzed by terminal deoxynucleotidyl transferase (TdT). The percentage of TUNEL‐positive cells was determined by flow cytometry (Beckman CytoFLEX), analyzing 10000 cells per sample, with data processed using FlowJo_V10 software.

### TUNEL Assay for HeLa Cells

HeLa cells were seeded into a 24‐well plate and transfected with an indicated plasmid for 24 h. Then, cells were collected by 0.25% Trypsin and washed with PBS. Next, the collected cells were fixed, incubated with PBS containing 0.3% Triton X‐100 for 5 min, and stained using the One Step TUNEL Apoptosis Assay Kit. Flow cytometry (Beckman, CytoFLEX) was used to detect the fluorescence intensity. 10 000 cells were gathered for each sample and analyzed by FlowJo_V10. Gating of the fixable Viability Dye negative cells to select positive cells.

### CASPASE 3 Activity and Apoptosis Detection for Live Cells

The CASPASE 3 Activity and Apoptosis Detection Kit for Live Cell was used to do this experiment. In short, the treated cells transfected with the indicated plasmid were incubated with a staining solution containing Annexin V‐mCherry and 1 mm GreenNuc CASPASE 3 Substrate for 30 min. Images were acquired via a high‐speed rotary disc‐type fluorescence confocal microscope (Andor Revolution‐XD, UK).

### RNA‐Seq Experiment

Whole transcriptome sequencing was conducted at Sangon Biotech (Shanghai, China). Following TRIzol‐mediated total RNA isolation from the cells, the RNA was examined for signs of degradation and contamination on 1% agarose gels, determined the RNA's purity using a NanoPhotometer spectrophotometer (Implen), and evaluated its integrity with a Bio‐analyzer 2100 system. The sequencing procedures were carried out in the same way as previously reported. Magnetic beads with Oligo(dT) attached were utilized to purify mRNA. The procedures of cDNA synthesis, end repair, A‐base addition, and ligation of the Illumina‐indexed adaptors were carried out in accordance with the provided instructions. The final library was made by denaturing and circularizing the double‐stranded PCR products from the previous step with the splint oligo sequence. The final library was prepared by formatting the single‐strand circular DNA (ssCir DNA). In order to create the DNA nanoball (DNB), the final library was amplified using phi29. In this study, the BGIseq500 platform was used to obtain single‐end 50‐base reads from DNBs placed into a patterned nanoarray. DESeq2 was used for the differential expression analysis and a threshold was set to a Q value with a false discovery rate (FDR) < 0.05.

### Transfection

The Lipofectamine 3000 Reagent was utilized for the purpose of transfecting DNA into HeLa cells. The cells were seeded into 12‐well plates or 24‐well plates with a density of 2 × 10^5^ cells per well. On the following day, the cells were subjected to transfection using plasmid DNA.

### HeLa Cell Infection

Grown‐overnight *Yptb* strains were cultured in YLB at 30 °C with appropriate antibiotics. The next day, the pellets were collected and resuspended in PBS. HeLa cells were grown in DMEM devoid of FBS and penicillin‐streptomycin prior to infection. HeLa cells were infected with *Yptb* strains at an MOI of 100. After centrifuging at 500 g for 5 min to bring the bacteria closer to the cells, the infected cells were incubated at 37 °C for 2 h. Then the cells were washed twice with PBS and resuspended in FBS‐ and penicillin‐ and streptomycin‐containing media and cultured at 37 °C in 5% CO_2_ for 2 h. At a total of 4 h’ infection, cells were taken out for subsequent study.

### Hochest 33 342/Propidium Iodide (PI) Assay

The Apoptosis and Necrosis Assay Kit was used to detect the form of cell death according to the manufacturer's protocol. In short, 5 × 10^5^ HeLa cells transfected with the specified plasmid were collected and washed twice with PBS solution. Cells were suspended in 100 µl of Binding Buffer and then treated with Hochest 33 342 and PI Staining Solution for 30 min on ice. Flow cytometry (Beckman, CytoFLEX) was used to examine the cell's condition.

### Annexin V‐FITC/Propidium Iodide (PI) Assay

Annexin V‐FITC/PI Apoptosis Detection Kit was used to detect cell apoptosis following the manufacturer's protocol. Briefly, a total of 5 × 10^5^ HeLa cells transfected with the indicated plasmid were collected and washed twice with PBS buffer. Cells were resuspended in 100 µL 1 × Binding Buffer and incubated with Annexin V‐FITC and PI Staining Solution for 10 min in the dark. Flow cytometry (Beckman, CytoFLEX) was utilized to analyze the apoptotic cells.

### Cell Cycle Analysis

The distribution of treated HeLa cells in the cell cycle phases was determined by measuring DNA content using DNA Content Quantitation Assay. In brief, treated cells were collected by 0.25% trypsin and fixed with the ice‐cold 70% ethanol overnight at 4 °C. Subsequently, cells were centrifugated to recover and washed with PBS. Finally, cells were incubated with RNase for 30 min at 37 °C and stained with PI for 30 min at 4 °C. Flow cytometry (Beckman, CytoFLEX) and software ModFit LT 5.0 were utilized to perform the cell cycle analysis.

### Statistical Analysis

Statistical analyses were performed using GraphPad Prism Software (GraphPad Prism 8.0.1). Statistical analyses in mice were analyzed using the Mann‐Whitney test. *P* values for mice survival were calculated using the Log‐rank test. All other experiments were analyzed using unpaired, two‐tailed Student's t test, one‐way or two‐way ANOVA test. Error bars indicate ± SD. Statistical significance is denoted in figures by asterisks. ^*^ *P *< 0.05; ^**^ *P *< 0.01; ^***^ *P *< 0.001.). The sample size (n) for each statistical analysis was indicated in the caption of each figure.

## Conflict of Interest

The authors declare no conflict of interest.

## Supporting information



Supporting Information

## Data Availability

All data are available in the main text or the supplementary materials. The 16S rRNA gene sequencing data have been deposited at the National Center for Biotechnology Information GenBank repository and China National Microbiology Data Center, and are publicly available as of the date of publication. Raw FASTQ files for the RNA‐seq libraries have been deposited at the NCBI Sequence Read Archive (SRA). Accession numbers are listed in the key resources table. This paper does not report original codes. Any additional information required to reanalyze the data reported in this paper is available from the lead contact upon request, Xihui Shen (xihuishen@nwsuaf.edu.cn).
