# Supplementary material for: A Dual‐Targeting T6SS DNase Drives Bacterial Antagonism and Eukaryotic Apoptosis via the cGAS‐STING‐TNF Axis
Source: Adv Sci (Weinh). 2025 May 14;12(28):2504086. doi: 10.1002/advs.202504086 (PMC12302616; doi:10.1002/advs.202504086)
Supplement: Supplementary file 1 — Supporting Information [file ADVS-12-2504086-s001.docx]

Supporting Information

A dual-targeting T6SS DNase drives bacterial antagonism and eukaryotic apoptosis via the cGAS-STING-TNF axis

*Li Song^1,2†^, Lei Xu^2†^, Pengfei Zhang^2^, Shuying Li^2^, Yichen Qu^2^, Yixin Zhao^2^, Zhenkun Shi^2^, Ruiqi Ma^2^, Yongdong Li^5^, Yi Chen^5^, Yao Wang^2^, Zhengfan Jiang^3,4^, Gehong Wei^1,2*^, Xihui Shen^2*^*

^1^Shaanxi Key Laboratory of Agricultural and Environmental Microbiology, College of Natural Resources and Environment, Northwest A&F University, Yangling, Shaanxi 12100, P. R. China;

^2^College of Life Sciences, Northwest A&F University, Yangling, Shaanxi 712100, P. R. China;

^3^Key Laboratory of Cell Proliferation and Differentiation of the Ministry of Education, School of Life Sciences, Peking University, Beijing, China.

^4^Peking-Tsinghua Center for Life Sciences, Peking University, Beijing, China.

^5^Ningbo Municipal Center for Disease Control and Prevention, Ningbo, Zhejiang 315010, P. R. China;


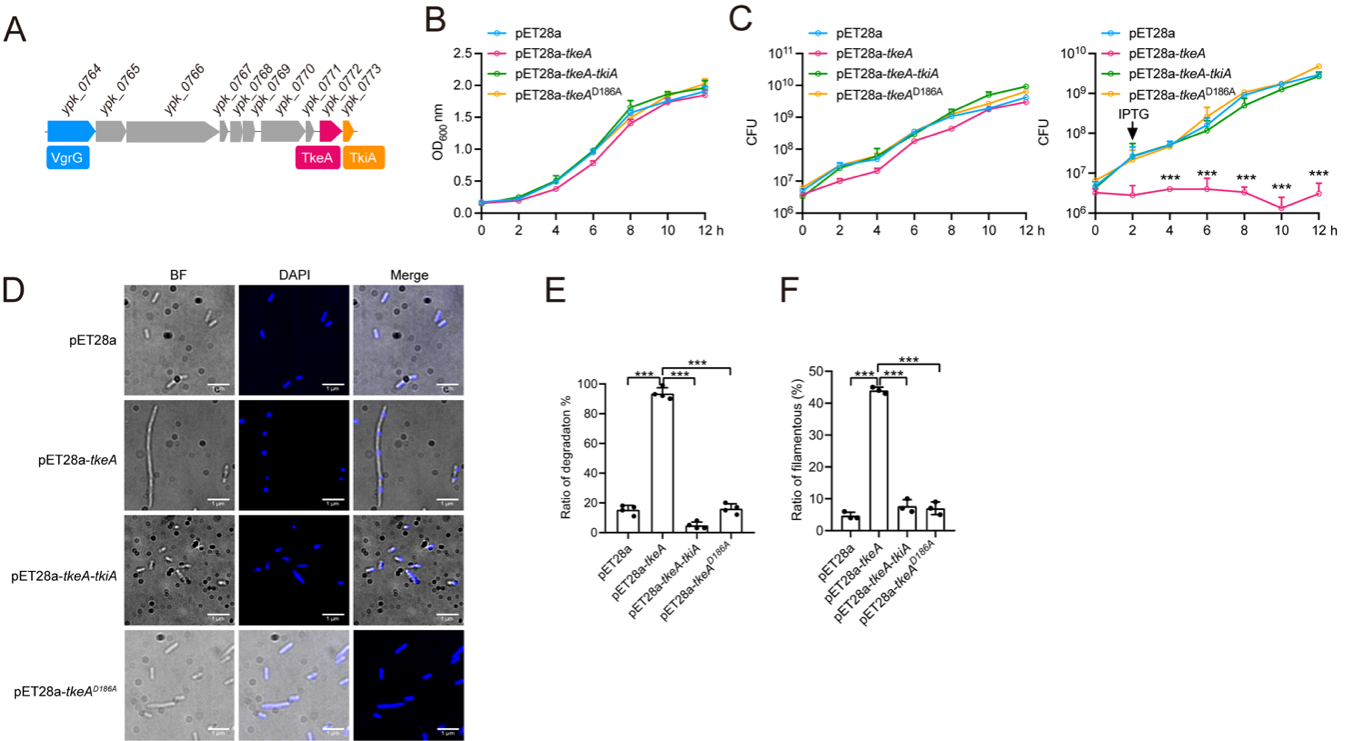


Figure S1. TkeA degrades *E. coli* genome DNA. Related to Figure 1

(A) Structure of the *Yptb* orphan gene cluster (From *ypk_0764* to *ypk_0773*). The *vgrG* gene (*ypk_0764*), *tkeA* gene (*ypk_0772*) and *tkiA* gene (*ypk_0773*) are colored in blue, yellow and red, respectively.

(B) Growth curves of *E. coli* BL21(DE3) containing indicated plasmids were determined by measuring OD_600_ from 0 h to 12 h at a 2 h interval.

(C) Cell viability of *E. coli* BL21(DE3) containing indicated plasmids was determined by measuring CFU from 0 h to 12 h at a 2 h interval.

(D) Detection of the loss of DNA staining (DAPI) in *E. coli* cells expression TkeA, TkeA^D186A^ and co-expressing TkeA-TkiA at 4 h after IPTG induction. Fluorescence microscopy was performed to visualize the genome degradation. Scale bar, 1 μm.

(E) and (F) The quantification in (D) of the degradation and filamentous were calculated. Data are from 3 biological replicates.

*P*  values calculated using one-way analysis of variance (ANOVA) for multiple comparisons.

Error bars represent ± SD. ****P*< 0.001.


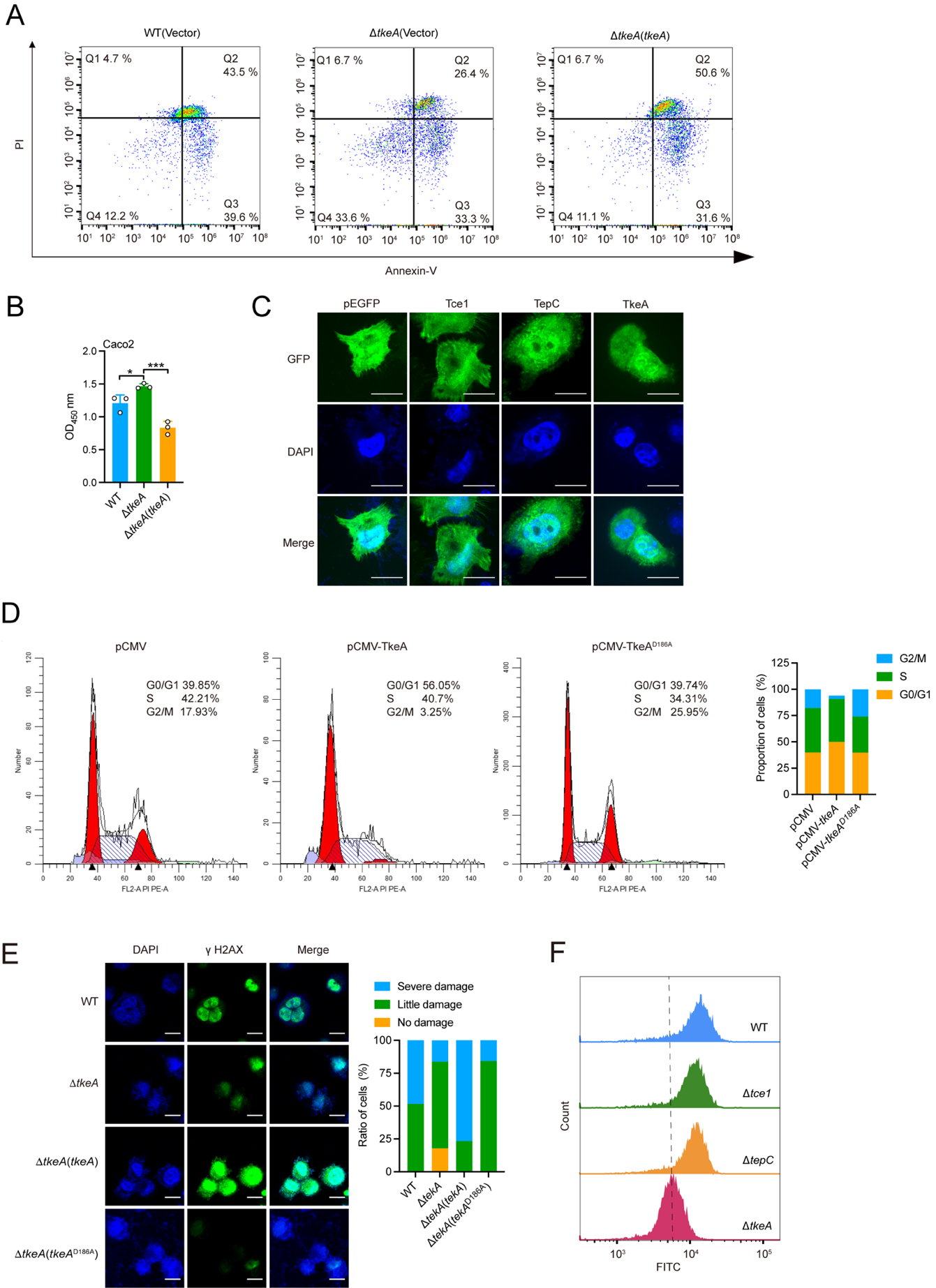


Figure S2. TkeA induces cell cycle arrest and apoptosis. Related to Figure 2

(A) Annexin V-FITC/PI staining of Caco-2 cells infected with *Yptb* WT, Δ*tkeA*, and Δ*tkeA*(*tkeA*) for 4 h (MOI = 100). n = 3.

(B) The cell counting assay of the *Yptb* WT, Δ*tkeA* and Δ*tkeA*(*tkeA*) infected Caco2 cells. Caco2 cells were incubated with relevant *Yptb* strains at a MOI of 100 for 1.5 h. 10 μL CCK-8 was added and incubated for 4 h. Absorbance was tested at 450 nm. n = 3.

(C) Representative immunofluorescence of GFP) in HeLa cells to show the distribution of these proteins. HeLa cells transfected with pEGFP, or pEGFP-*tce1*, pEGFP-*tepC* and pEGFP-*tkeA* for 24 h. Scale bar, 500 μm.

(D) HeLa cells were transfected with pCMV (Control), pCMV-*tkeA* vector and pCMV-*tkeA^D186A^* and were collected and stained with PI. The cell cycle was detected by flow cytometry.

Right panel: The quantification in (D) of the cell cycle.

(E) Detection of DNA damage led by TkeA in HeLa cell. HeLa cells were infected with *Yptb* WT, Δ*tkeA*, Δ*tkeA*(*tkeA*) and Δ*tkeA*(*tkeA*^D186A^) for 4 h. Nuclear DNA was stained with DAPI (blue). γ-H2AX signal was examined using immunofluorescence microscopy (green). The quantification was calculated on the right. Scale bar, 500 μm.

(F) Detection of TkeA, Tce1 and TepC-induced genomic DNA fragmentation after 4 h IPTG induction in the TUNEL assay. DNA fragmentation was detected based on monitoring of fluorescence intensity (indicated on the X-axis) using flow cytometry. The counts resulting from cell sorting are indicated on the Y-axis.

*P*  values calculated using one-way analysis of variance (ANOVA) for multiple comparisons.

Error bars represent ± SD. **P* < 0.05; ****P* < 0.001.


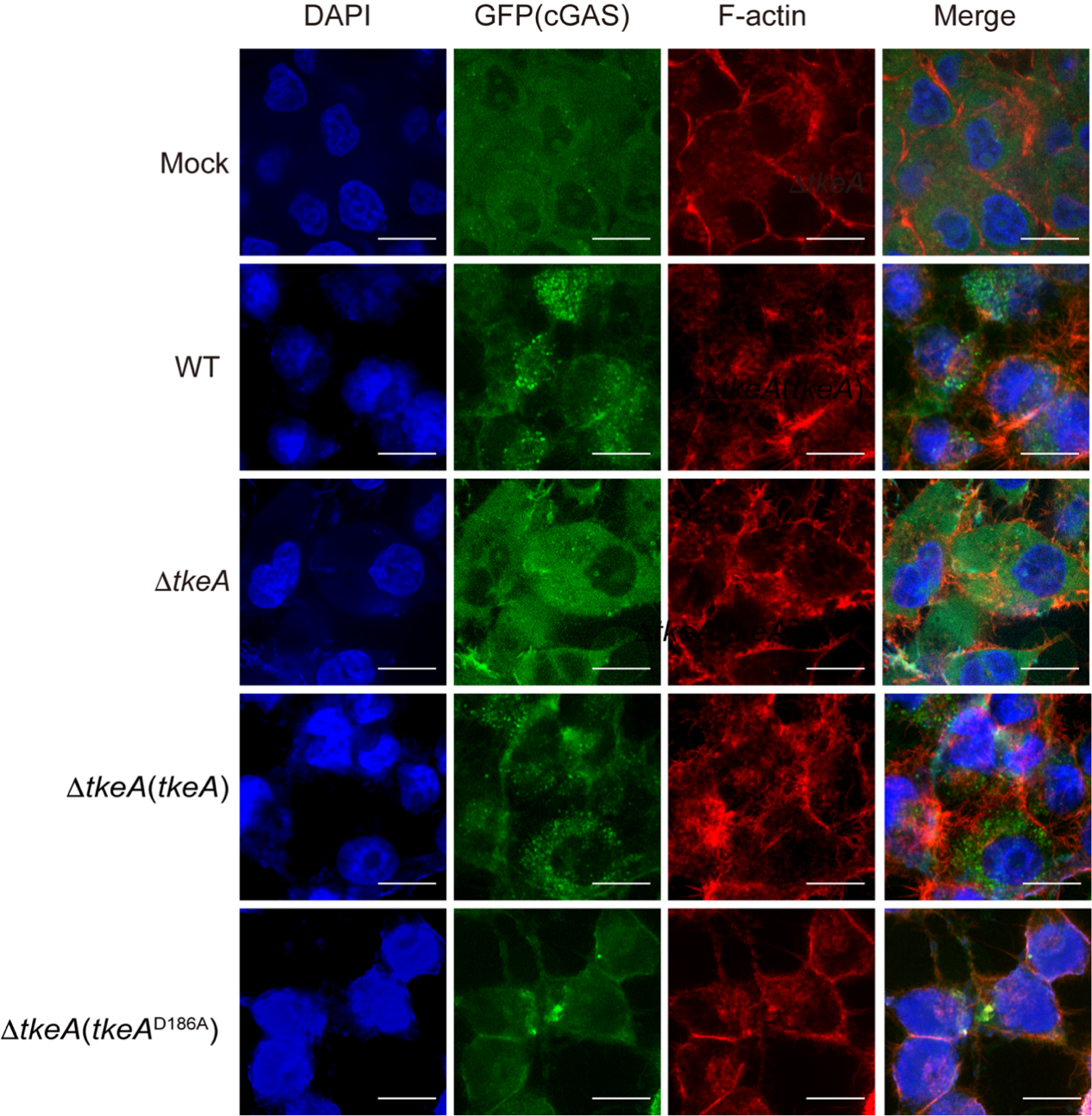


Figure S3. TkeA activates the cGAS-STING pathway. Related to Figure 3

Translocation of TkeA and activation cGAS. HeLa cells were infected with *Yptb* WT, Δ*tkeA*, Δ*tkeA*(*tkeA*) and Δ*tkeA*(tkeA^D186A^) for 4 h. Fluorescence microscopy was performed to visualize the activation of cGAS. DAPI, nucleus; GFP, cGAS; F-actin, cytoskeleton. Scale bar, 500 μm.


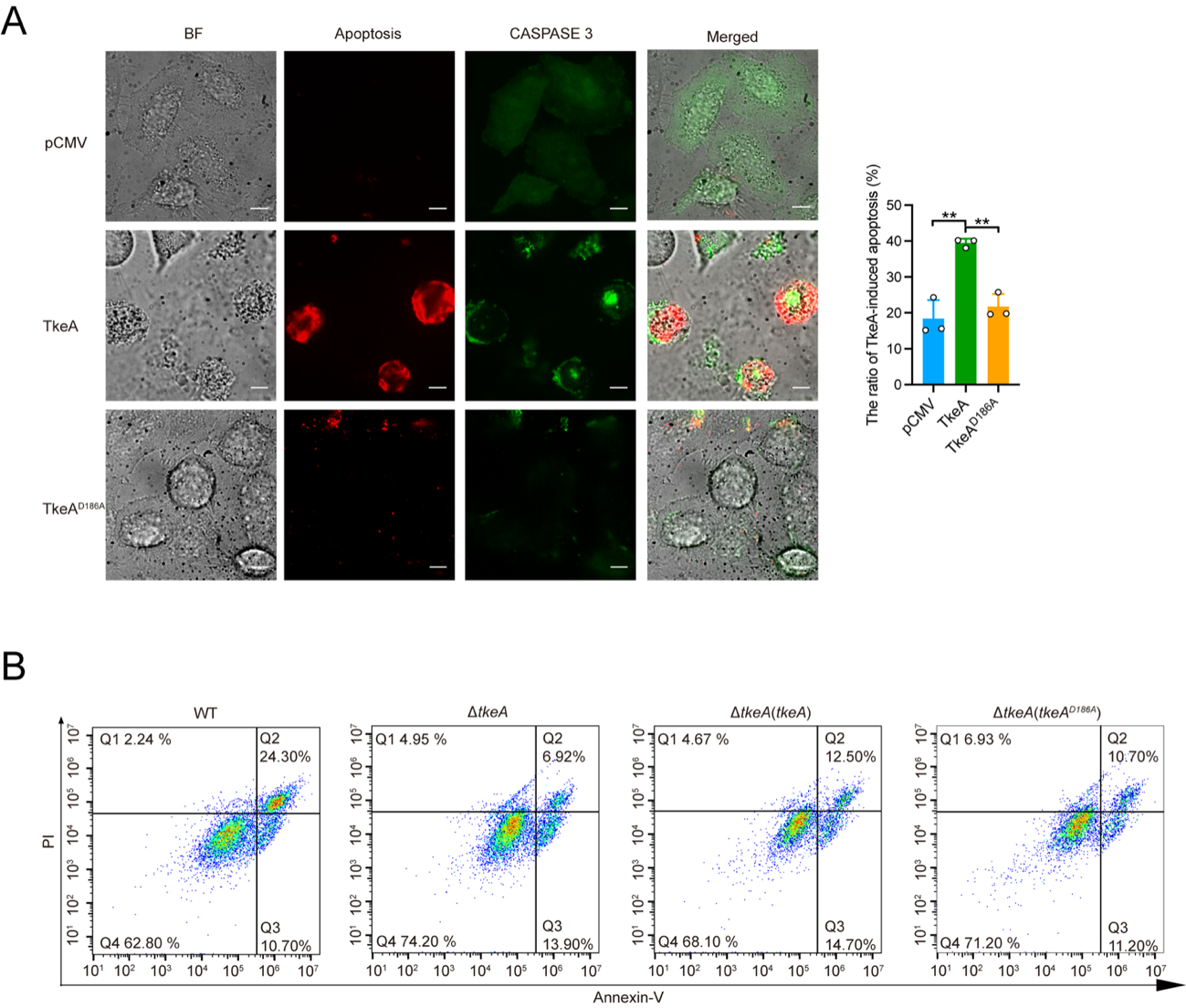


Figure S4. TkeA activates CASPASE 3 and apoptosis in host cells. Related to Figure 4

(A) CASPASE 3 activated and apoptosis test in HeLa cells. HeLa cells transfected with pCMV, pCMV-*tkeA* and pCMV-*tkeA^D186A^* for 24 h were stained with GreenNuc™ CASPASE 3 and Annexin V-mCherry. Images are representative cells from the same field of view. Fluorescence microscopy was performed to visualize the activation of CASPASE 3 and apoptosis. Scale bar, 500 μm. The right panel is the quantification of the ratio of TkeA-induced apoptosis.

(B) HeLa cells infected with the indicated *Yptb* strains were collected and stained with Annexin V/PI. Flow cytometry was used to identify the cell apoptosis.


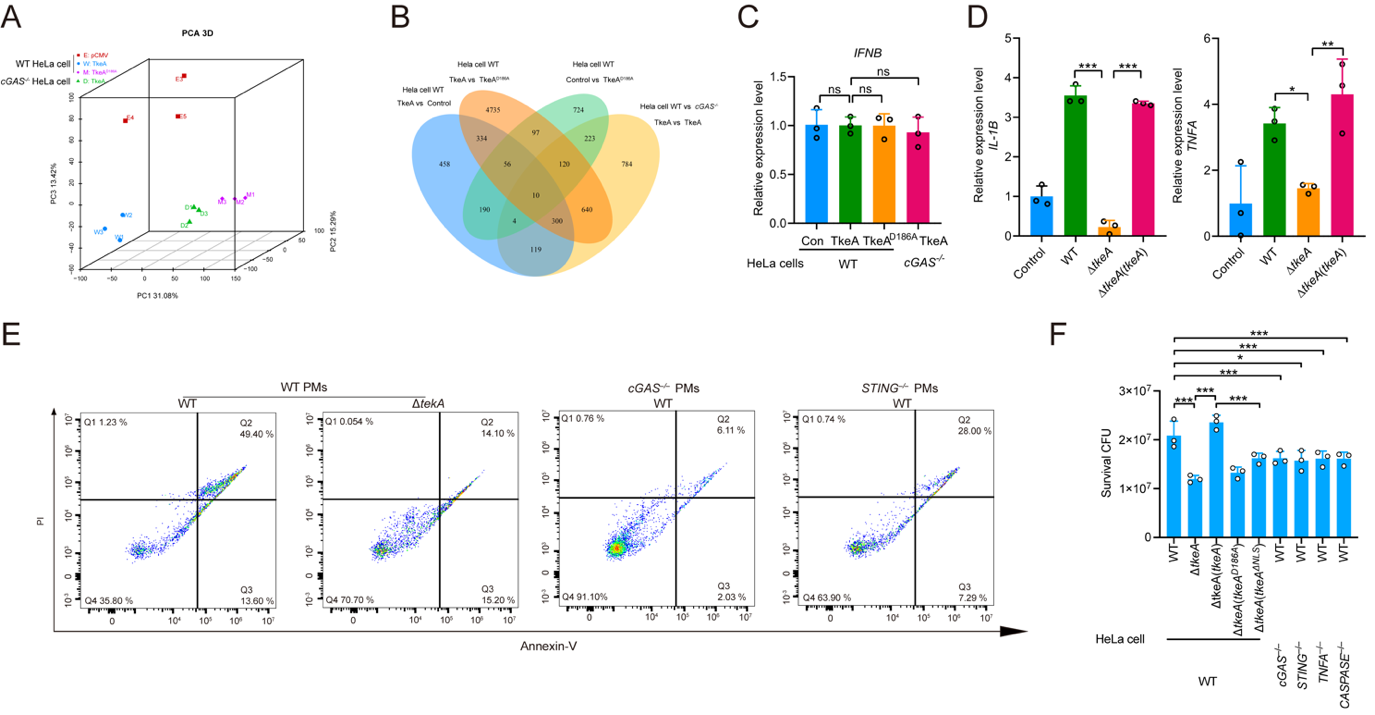


Figure S5. The cGAS-STING-TNF signaling pathway is implicated in TkeA-induced apoptosis. Related to Figure 5

(A) WT HeLa cells transfected with pCMV, pCMV-*tkeA* and pCMV-*tkeA^D186A^*, and *cGAS*^–/–^ HeLa cells transfected with pCMV-*tkeA*. RNA isolated from these cells was subject to RNA-seq. Principal component analysis (PCA) was used to compare the four groups. n = 3.

(B) A Venn diagram was used to illustrate the unions, intersections and distinctions among four groups. n = 3.

(C) qRT-PCR analysis of gene expression in WT HeLa cells transfected with pCMV, pCMV-*tkeA* and pCMV-*tkeA^D186A^*, and cGAS^–/–^HeLa cells transfected with pCMV-*tkeA*. n = 3.

(D) qRT-PCR analysis of gene expression in WT HeLa cells infected with *Yptb* WT, Δ*tkeA* and Δ*tkeA(tkeA)* for 4 h at an MOI of 100. n  =  3.

(E) C57BL/6 wild-type, *cGas*^–/–^ and *Sting*^–/–^ mouse PMs infected with *Yptb* WT and Δ*tkeA* for 4 h at an MOI of 100. Cells were collected and stained with Annexin V/PI. Flow cytometry was used to identify cell apoptosis.

(F) Survival of *Yptb* WT, Δ*tkeA*, and Δ*tkeA*(*tkeA*) in WT, *cGAS*^–/–^, *STING*^–/–^, *TNFA*^–/–^and *CASPASE* 3^–/–^ HeLa cells after 4 h infection (MOI = 100). n = 3

*P*  values calculated using one-way ANOVA for multiple comparisons.

Error bars represent ± SD. **P*< 0.05; ****P*< 0.001. ns, not significant.


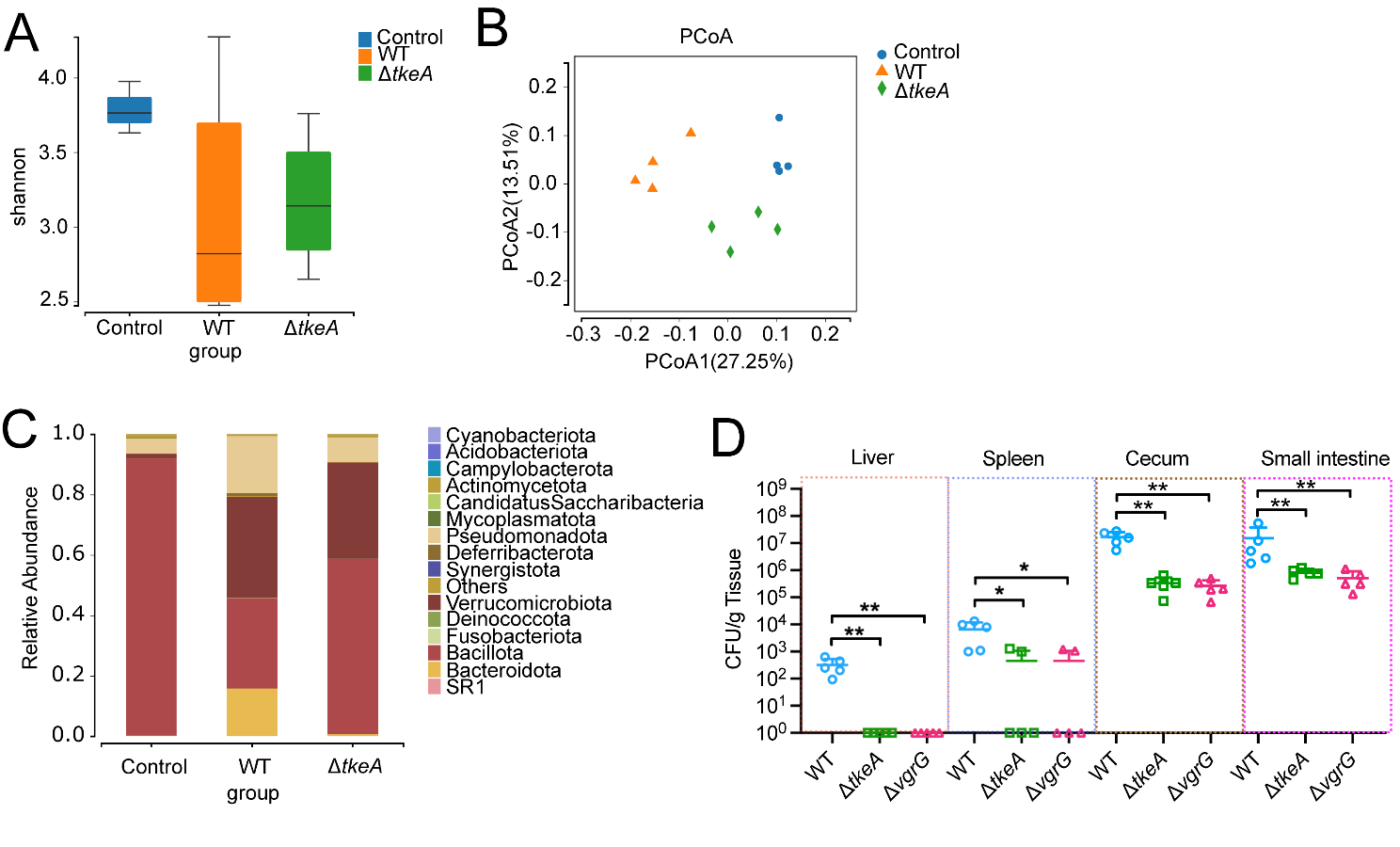


Figure S6. The analysis of the 16S rRNA gene amplicon of the microbiota of mice infected with different *Yptb* strains. Related to Figure 6

(A) Mice were orally gavaged with 10^9^ CFUs of different *Yptb* strains. Alpha diversity of the gut microbiota with the Shannon index in the three groups. The horizontal bars within boxes represent medians.

(B) CPCoA with Bray-Curtis distance showing the beta diversity (*P* <0.001, PERMANOVA by Adonis).

(C) Phylum-level distribution of the native gut microbiota in three groups.

(D) Mice were pretreated with antibiotics and orally gavaged with 10^9^ CFUs of different *Yptb* strains. Homogenates of the liver, spleen, cecum and small intestine were plated to determine the bacterial CFU counts per gram of organs at 24 h post-infection. n = 5

*P*  values in (D) calculated using the Mann-Whitney test.

Table S1 Bacterial strains and plasmids used in this study.

| **Strain or plasmid** | **Relevant characteristics** | **Reference** |
| --- | --- | --- |
| ***E. coli*** |  |  |
| S17-1*λ pir* | *λ*-pir lysogen of S17-1, *thi pro hsdR hsdM^+^ recA* RP4 2-Tc::Mu-Km::Tn7 | 1 |
| BL21(DE3) | Host for expression vector pET28a and toxin assay | Novagen |
| BTH101 | Host for bacterial two-hybrid | Novagen |
| *DH5α* | competitor for competition assay | Beyotime |
|  |  |  |
| ***Yersinia pseudotuberculosis* Ⅲ** |  |  |
| WT | Wild-type *Y. pseudotuberculosis* pIB1, Nal^r^ | 2 |
| Δ*vgrG* | *vgrG* gene deleted in *Y. pseudotuberculosis* | This study |
| Δ*vgrG* (*vgrG*) | *vgrG* gene complemented in *Y. pseudotuberculosis* Δ*vgrG* | This study |
| Δ*clpV1* | *clpV1* gene deleted in *Y. pseudotuberculosis* | 2 |
| Δ*clpV2* | *clpV2* gene deleted in *Y. pseudotuberculosis* | 2 |
| Δ*clpV3* | *clpV3* gene deleted in *Y. pseudotuberculosis* | 2 |
| Δ*clpV4* | *clpV4* gene deleted in *Y. pseudotuberculosis* | 2 |
| Δ*4clpV* | *clpV1*, *clpV2*, *clpV3*, *clpV4* gene deleted in *Y. pseudotuberculosis* | 2 |
| Δ*tkeA* | *tkeA* gene deleted in *Y. pseudotuberculosis* | This study |
| Δ*tkeA*Δ*tkiA* | *tkeA* and *tkiA* gene deleted in *Y. pseudotuberculosis* | This study |
| Δ*tkeA*Δ*tkiA*(*tkiA*) | *tkiA* gene complemented in *Y. pseudotuberculosis* Δ*tkeA*Δ*tkiA* | This study |
| Δ*tkeA*(*tkeA^D186A^*) | *tkeA^D186A^* gene complemented in *Y. pseudotuberculosis* Δ*tkeA* | This study |
|  |  |  |
| ***Salmonella* Typhimurium** |  |  |
| *SL1344* | competitor for competition assay | Laboratory |
|  |  |  |
| **Plasmid** |  |  |
| pME6032 | Shuttle vector, Tc^r^ | 3 |
| pME6032-*tkeA-vsvg* | *tkeA-vsvg* under the control of chloramphenicol resistance gene promoter in plasmid pME6032 | This study |
| pME6032-*tkeA-tem* | *tkeA-tem* under the control of chloramphenicol resistance gene promoter in plasmid pME6032 | This study |
| pET28a | Expression vector with N-terminal hexahistidine affinity tag, Km^r^ | Novagen |
| pET28a-*tkeA* | pET28a carrying *tkeA* coding region, Km^r^ | This study |
| pET28a-*tkeA-tkiA* | pET28a carrying *tkeA and tkiA* coding region, Km^r^ | This study |
| pET28a-*tkeA^D186A^* | pET28a carrying *tkeA^D186A^* coding region, Km^r^ | This study |
| pDM4 | Suicide vector, *mob*RK2, *ori*R6K, *pir*, *sacB*, Cm^r^ | 4 |
| pDM4-Δ*tkeA* | Construct used for in-frame deletion of *tre1,* Cm^r^ | This study |
| pDM4-Δ*tkeA*Δ*tkiA* | Construct used for in-frame deletion of *tre1* and *tri1,* Cm^r^ | This study |
| pDM4-Δ*clpV1* | Construct used for in-frame deletion of *clpV1*, Cm^r^ | This study |
| pDM4-Δ*clpV2* | Construct used for in-frame deletion of *clpV2*, Cm^r^ | This study |
| pDM4–Δ*clpV3* | Construct used for in-frame deletion of *clpV3*, Cm^r^ | This study |
| pDM4–Δ*clpV4* | Construct used for in-frame deletion of *clpV4*, Cm^r^ | This study |
| pKT100 | Cloning vector, p15A replicon, Km^r^ | 5 |
| pKT100-*clpV1* | *clpV1* under the control of chloramphenicol resistance gene promoter in plasmid pKT100 | This study |
| pKT100-*clpV2* | *clpV2* under the control of chloramphenicol resistance gene promoter in plasmid pKT100 | This study |
| pKT100-*clpV3* | *clpV3* under the control of chloramphenicol resistance gene promoter in plasmid pKT100 | This study |
| pKT100-*clpV4* | *clpV4* under the control of chloramphenicol resistance gene promoter in plasmid pKT100 | This study |
| pKT100-*tkeA* | TkeA under the control of chloramphenicol resistance gene promoter in plasmid pKT100 | This study |
| pKT100-*tkeA^D186A^* | TkeA^D186A^ under the control of chloramphenicol resistance gene promoter in plasmid pKT100 | This study |
| pKT100-*vgrG* | *vgrG* under the control of chloramphenicol resistance gene promoter in plasmid pKT100 | This study |
| pKT25 | p15A origin of replication encoding CyaA_1224_; Km^r^ | 6 |
| pUT18C | ColE1 origin of replication encoding CyaA_225399_; Amp^r^ | 6 |
| pKT25-*tkeA* | *tkeA* in pKT25 | This study |
| pUT18C-*tkiA* | *tkiA* in pUT18C | This study |
| pUT18C-*vgrG* | *vgrG* in pUT18C | This study |
| pKT25*-zip* | Leucine zipper of GCN1 (BTH positive control); Km^r^ | 6 |
| pUT18C-*zip* | Leucine zipper of GCN1 (BTH positive control); Amp^r^ | 6 |
| pEGFP | Cloning and expressing vector, Amp^r^ | This study |
| pEGFP-*tkeA* | *tkeA* in pEGFP | This study |
| pEGFP-*tkeA^D186A^* | *tkeA^D186A^* in pEGFP | This study |
| pCMV | Cloning and expressing vector, Km^r^ | This study |
| pCMV-*tkeA* | *tkeA* in pCMV | This study |
| pCMV-*tkeA^D186A^* | *tkeA^D186A^* in pCMV | This study |
| pCMV-C-EGFP | EGFP in pCMV | This study |
| pCMV-*cgas-gfp* | *cGas-gfp* in pCMV | This study |

* Nal^R^, Cm^R^, Km^R^, Tc^R^, Gm^R^, and Amp^R^ represent resistance to Nalidixic acid, chloramphenicol, kanamycin, tetracycline and ampicillin, respectively.

Table S2 Primers used in this study.

| **Primers** | **5’-3’ sequence** | **Function** |
| --- | --- | --- |
| *tkeA-*M1F | GGTTACCCGCATGCAAGATCTATCTAGAATGAGGATTTTTTAATGATTGAA | To generate  pDM4-Δ*tkeA* and  pDM4-Δ*tkeA*Δ*tkiA* |
| *tkeA-*M1R (*tkiA*) | CTATAACCCGATGCTTTTTAGCACAGCGGATGGTTCTG |  |
| *tkeA-*M1R (*tkeA*) | TCATGCATCCCCCATTCACAGCGGATGGTTCTG |  |
| *tkeA-*M2F | CAGAACCATCCGCTGTGAATGGGGGATGCATGA |  |
| *tkeA-*M2R | ATATCAAGCTTATCGATACCGTCGACGCTTTTTAGTTCAATCTCAGAA |  |
| *tkiA-*M2F | CAGAACCATCCGCTGTGCTAAAAAGCATCGGGTTATAG |  |
| *tkiA-*M2R | ATATCAAGCTTATCGATACCGTCGACCCCGTAGTCCCGTAGTCC |  |
| *vgrG-*M1F | GGTTACCCGCATGCAAGATCTATCTAGAAGAAGGAAATCGCCAGTAAA | To generate  pDM4-Δ*vgrG* |
| *vgrG-*M1R | TACGTTAAGGATTTAATTTTAGAATTTTATTACTGTCAGCAAACATATCT |  |
| *vgrG-*M2F | AGATATGTTTGCTGACAGTAATAAAATTCTAAAATTAAATCCTTAACGTA |  |
| *vgrG-*M2R | ATATCAAGCTTATCGATACCGTCGACAGAGATCCGCGGTTAATG |  |
| *tkeA-*F-*Spe*I | GGACTAGTATGCCAGAACCATCCGCT | To generate  pKT100-*tkeA* |
| *tkeA-*R-*Sal*I | ACGCGTCGACTCATTTTTTCATGCATCCC |  |
| *tkeA^D186A^-*F | CAAGCTCGTGGCTATGCGCAGTTTTATATTGATG | To generate  pET28a-*tkeA^D186A^*, pKT100-*tkeA^D186A^,* pEGFP-*tkeA^D186A^*  and pCMV-*tkeA^D186A^* |
| *tkeA^D186A^-*R | CATCAATATAAAACTGCGCATAGCCACGAGCTTG |  |
| *tkiA-*F-*Spe*I | GGACTAGTATGATTAAGAAAAAGAAAAAAAACAAT | To generate  pKT100-*tkiA* |
| *tkiA-*R-*Sal*I | ACGCGTCGACCTATAACCCGATGCTTTTTAGT |  |
| *vgrG-*F*-Bam*HI | CGCGGATCCATGAGTGATTTTAAATTTTGGCGTT | To generate  pKT100-*vgrG* |
| *vgrG-*R*-Sal*I | ACGCGTCGACGCTTAAGGATTTAATTTTAGAATGCC |  |
| *tkeA-*F-*Eco*RI | CCGGAATTCATGAAACCGTTAGATCAGAATATTACATAT | To generate  pME6032-*tkeA*-*vsvg* |
| *tkeA-*R*-Bgl*II | GAAGATCTTCATTTTCCTAATCTATTCATTTCAATATCTGTATAACATCCTCCGCCACCTTTT |  |
| *tkeA-*F-*Eco*RI | CCGGAATTCATGAAACCGTTAGATCAGAATATTACATAT | To generate  pEGFP-*tkeA* |
| *tkeA-*R-*Sal*I | ACGCGTCGACTCATTTTTTCATGCATCCC |  |
| *tkeA-*F*-Sal*I | ACGCGTCGACATGCCAGAACCATCCGCT | To generate  pCMV-*tkeA-flag* |
| *tkeA-*R*-Xba*I | CCGCTCGAGTCATTTTTTCATGCATCCC |  |
| *cgas*-F-*Hin*dIII | CCCAAGCTTATGCAGCCTTGGCACGGAAA | To generate  pCMV-*cgas*-*gfp* |
| *cgas*-R-*Bgl*II | GGAAGATCTAAATTCATCAAAAACTGGAAACTCATTG |  |
| *tkeA-*F*-Eco*RI | CGGAATTCATGCCAGAACCATCCGCT | To generate pET28a-*tkeA* |
| *tkeA-*R-*Sal*I | ACGCGTCGACTCATTTTTTCATGCATCCC |  |
| *tkeA-tkiA-*F*-Eco*RI | CGGAATTCATGCCAGAACCATCCGCT | To generate pET28a-*tkeA-tkiA* |
| *tkeA-tkiA-*R-*Sal*I | ACGCGTCGACCTATAACCCGATGCTTTTTAGT |  |
| *tkeA-*F*-Xba*I | GCTCTAGAATGCCAGAACCATCCGCT | To generate  pKT25-*tkeA* |
| *tkeA-*R*-Eco*RI | CGGAATTCTCATTTTTTCATGCATCCC |  |
| *tkiA-*F*-Xba*I | GCTCTAGAATGATTAAGAAAAAGAAAAAAAACAAT | To generate pUT18C-*tkiA* |
| *tkiA-*R*-Eco*RI | CGGAATTCCTATAACCCGATGCTTTTTAGT |  |
| *vgrG-*F*-Xba*I | GCTCTAGAATGTTTGCTGACAGTAATAAAGC | To generate pUT18C-*vgrG* |
| *vgrG-*R*-Kpn*I | GGGGTACCTTAAGGATTTAATTTTAGAATGCC |  |
| *tkeA-*F-*Eco*RI | GATAACAATTTCACACAGGAAACAGAATTCATGCCAGAACCATCCGCT | To generate pME6032-*tkeA*-*tem* |
| *tkeA-*R*-Bgl*I | CTGATCCGCTAGTCCGAGGCCTCGAGATCTTCATTTTTTCATGCATCCC |  |
| 16S_EC_-F | CTCACCAAGGCGACGAT | For qPCR |
| 16S_EC_-R | CCGCCAGCGTTCAATC |  |
| *RECA*-F | CGGTGAACTGGTTGACTT |  |
| *RECA*-R | GCATTGGCTTTACCCTGA |  |
| *LEXA*-F | ACCCATCCCTGTTCAAGC |  |
| *LEXA*-R | TGTACCGCCAACAAGTCA |  |
| *IL1Β*-F | CCACAGACCTTCCAGGAGAATG |  |
| *IL1Β*-R | GTGCAGTTCAGTGATCGTACAGG |  |
| *TNFΑ-*F | CTCTTCTGCCTGCTGCACTTTG |  |
| *TNFΑ*-R | ATGGGCTACAGGCTTGTCACTC |  |
| *NLRP3*-F | GGACTGAAGCACCTGTTGTGCA |  |
| *NLRP3*-R | TCCTGAGTCTCCCAAGGCATTC |  |
| *IL6*-F | AGACAGCCACTCACCTCTTCAG |  |
| *IL6*-R | TTCTGCCAGTGCCTCTTTGCTG |  |
| *GAPDH*-F | GTCTCCTCTGACTTCAACAGCG |  |
| *GAPDH*-R | ACCACCCTGTTGCTGTAGCCAA |  |
| *ACTB*-F | CACCATTGGCAATGAGCGGTTC |  |
| *ACTB*-R | AGGTCTTTGCGGATGTCCACGT |  |
| *CXCL10*-F | GGTGAGAAGAGATGTCTGAATCC |  |
| *CXCL10*-R | GTCCATCCTTGGAAGCACTGCA |  |
| *CXCL11*-F | AAGGACAACGATGCCTAAATCCC |  |
| *CXCL11*-R | CAGATGCCCTTTTCCAGGACTTC |  |
| *IL2RG*-F | CACTCTGTGGAAGTGCTCAGCA |  |
| *IL2RG*-R | GAGCCAACAGAGATAACCACGG |  |
| *TNFAIP3*-F | CTCAACTGGTGTCGAGAAGTCC |  |
| *TNFAIP3*-R | TTCCTTGAGCGTGCTGAACAGC |  |
| *IL1Α*-F | TGTATGTGACTGCCCAAGATGAAG |  |
| *IL1Α*-R | AGAGGAGGTTGGTCTCACTACC |  |
| *BIRC3*-F | GCTTTTGCTGTGATGGTGGACTC |  |
| *BIRC3*-R | CTTGACGGATGAACTCCTGTCC |  |
| *CCL3L1*-F | ACTTTGAGACGAGCAGCCAGTG |  |
| *CCL3L1*-R | TTTCTGGACCCACTCCTCACTG |  |
| *FLT4*-F | TGCGAATACCTGTCCTACGATGC |  |
| *FLT4*-R | CTTGTGGATGCCGAAAGCGGAG |  |
| *CSF1*-F | TGAGACACCTCTCCAGTTGCTG |  |
| *CSF1*-R | GCAATCAGGCTTGGTCACCACA |  |
| *GH1*-F | TCTTCGCCAACAGCCTGGTGTA |  |
| *GH1*-R | GTCGAACTTGCTGTAGGTCTGC |  |
| *TNFSF13B*-F | ACCACGCGGAGAAGCTGCCAG |  |
| *TNFSF13B*-R | CTGCTGTTCTGACTGGAGTTGC |  |
| *CCL20*-F | AAGTTGTCTGTGTGCGCAAATCC |  |
| *CCL20*-R | CCATTCCAGAAAAGCCACAGTTTT |  |
| *TNFSF15*-F | CACCACATACCTGCTTGTCAGC |  |
| *TNFSF15*-R | TCTCCGTCTGCTCTAAGAGGTG |  |
| *TNFRSF9*-F | TCTTCCTCACGCTCCGTTTCTC |  |
| *TNFRSF9*-R | TGGAAATCGGCAGCTACAGCCA |  |
| *TRAF1*-F | CGATGGCACTTTCCTGTGGAAG |  |
| *TRAF1*-R | TACAGCCGCAGGCACAACTTGT |  |
| *CCL5*-F | CCTGCTGCTTTGCCTACATTGC |  |
| *CCL5*-R | ACACACTTGGCGGTTCTTTCGG |  |
| *Tnfa*-Mouse-F | GGTGCCTATGTCTCAGCCTCTT |  |
| *Tnfa*-Mouse-R | GCCATAGAACTGATGAGAGGGAG |  |
| *Casp8*-Mouse-F | ATGGCTACGGTGAAGAACTGCG |  |
| *Casp8*-Mouse-R | TAGTTCACGCCAGTCAGGATGC |  |
| *Casp3*-Mouse-F | GGAGTCTGACTGGAAAGCCGAA |  |
| *Casp3*-Mouse-R | CTTCTGGCAAGCCATCTCCTCA |  |

Underlined sites indicate restriction enzyme cutting sites added for cloning. Letters in boldface denote the annealing regions for overlap PCR.

Table S3 KEY RESOURCES TABLE

| REAGENT or RESOURCE | SOURCE | IDENTIFIER |
| --- | --- | --- |
| Chemicals, Peptides, and Recombinant Proteins | | |
| Tryptone | OXOID | #LP0021 |
| Yeast extract | OXOID | #LP0042 |
| NaCl | Ghtech | #7647-14-5 |
| Na_2_HPO_4_ | Ghtech | #10039-32-4 |
| KH_2_PO_4_ | Ghtech | #7778-77-0 |
| NH_4_Cl | Ghtech | #12125-02-9 |
| MgSO_4_ | Ghtech | #7487-88-9 |
| CaCl_2_ | Ghtech | #10043-52-4 |
| glucose | Ghtech | #50-99-7 |
| MgCl_2_ | Sigma-Aldrich | #M8266 |
| MacConkey Agar | Solarbio | #M8560 |
| o-nitrophenyl-β-D-galactopyranoside (ONPG) | Solarbio | #O8040 |
| nalidixic acid | Solarbio | #N8080 |
| kanamycin | Solarbio | #K8020 |
| ampicillin | Solarbio | #A6920 |
| chloramphenicol | Solarbio | #C8050 |
| tetracycline | Solarbio | #T8180 |
| streptomycin | Solarbio | #S8290 |
| Isopropyl β-D-Thiogalactopyranoside (IPTG) | Solarbio | #I8070 |
| Tris (Hydroxymethyl) Aminomethane | Solarbio | #T8060 |
| DAPI | Solarbio | #C0060 |
| TRIzol Reagent | Sigma-Aldrich | #T9424 |
| Lipofectamine 3000 Reagent | Invitrogen | #L3000015 |
| λ DNA | Takara | #3010 |
| MES | Solarbio | #M8010 |
| EDTA | Solarbio | #E8040 |
| Triton X-100 | Solarbio | #T8200 |
| Z-VAD-FMK(20 mM) | YESEN | #40328ES25 |
| Medium and solution | | |
| Phosphate-Buffered Saline (PBS) | Beyotime | #C0221A |
| Penicillin-Streptomycin Solution (100X) | Beyotime | #C0222 |
| Dulbecco's Modified Eagle Medium (DMEM) High glucose | GIBCO | #11965-084 |
| Opti-MEM I Reduced Serum Media | GIBCO | #11058-021 |
| Fetal Bovine Serum (FBS) | GIBCO | #10099-141 |
| QuickBlock™ Blocking Buffer | Beyotime | #P0252 |
| QuickBlock™ Primary Antibody Dilution Buffer for Western Blot | Beyotime | #P0256 |
| Antibodies | | |
| Rabbit monoclonal anti-His-Tag | Cell Signaling Technology | #12698 RRID: AB_2744546 |
| Rabbit monoclonal anti-p-IRF3 (Ser396) (clone 4D4G) | Cell Signaling Technology | #4947 RRID: AB_823547 |
| Rabbit polyclonal anti-β-actin | Cell Signaling Technology | #4967 RRID: AB_330288 |
| Rabbit polyclonal anti-cleaved Caspase-3 (Asp175) | Cell Signaling Technology | #9661 RRID: AB_2341188 |
| Rabbit polyclonal anti-cleaved Caspase-3 | Cell Signaling Technology | #9662 RRID: AB_331439 |
| Rabbit monoclonal anti-cGAS | Cell Signaling Technology | #15102 RRID: AB_2732795 |
| Rabbit monoclonal anti-Phospho  TBK1/NAK (Ser172) | Cell Signaling Technology | #5483 RRID: AB_10693472 |
| Rabbit polyclonal anti-TBK1/NAK | Cell Signaling Technology | #3013 RRID: AB_2199749 |
| Rabbit polyclonal anti-TNFα | Cell Signaling Technology | #3707 RRID: AB_2240625 |
| Rabbit polyclonal anti-Cleaved Caspase-8 (Asp374) (18C8) | Cell Signaling Technology | #9496 RRID: AB_561381 |
| Rabbit polyclonal anti-NF-κB p65(D14E12) | Cell Signaling Technology | #8242 RRID: AB_10859369 |
| Mouse Monoclonal anti-VSVG | Santa Cruz Biotechnology | #sc-365019 RRID: AB_10846802 |
| Mouse monoclonal anti-RNAP | Biolegend | #663104 RRID: AB_2687386 |
| HRP-labeled Goat Anti-Rabbit IgG (H+L) | Beyotime | #A0208 RRID: AB_2892644 |
| HRP-labeled Goat Anti-Mouse IgG (H+L) | Beyotime | #A0216 RRID: AB_2860575 |
| Experimental Models: Organisms/Strains | | |
| Mouse: BALB/c | Beijing Weitonglihua | N/A |
| Mouse: C57BL/6JNju | GemPharmatech | N/A |
| Mouse: *cGas*^–/–^ C57BL/6 | Dr. Zhengfan Jiang | N/A |
| Mouse: *Sting*^–/–^ C57BL/6 | Dr. Zhengfan Jiang | N/A |
| Experimental Models: Cell Lines | | |
| Human: WT HeLa cells | Dr. Zhengfan Jiang | N/A |
| Human: *cGAS*^–/–^ HeLa cells | Dr. Zhengfan Jiang | N/A |
| Human: *STING*^–/–^ HeLa cells | Dr. Zhengfan Jiang | N/A |
| Human: *CASPASE* *3*^–/–^ HeLa cells | ShareBio | SB-KO131 |
| Human: *CASPASE 8*^–/–^ HeLa cells | Guangdong Yuanjing | YKO-HS3744 |
| Human: *TNFA*^–/–^ HeLa cells | Guangdong Yuanjing | YKO-HS7531 |
| Commercial Assays | | |
| QuickMutation™ Random Mutagenesis Kit | Beyotime | #D0219M |
| QuickMutation™ Site-Directed Mutagenesis Kit | Beyotime | #D0206S |
| Cell Counting Kit-8 | Beyotime | #C0037 |
| Caspase-3 Activity and Mitochondrial Membrane Potential Detection Kit for Live Cell | Beyotime | #C1073S |
| Annexin V-FITC/PI Apoptosis Detection Kit | Vazyme | #A211-01 |
| Apoptosis and Necrosis Assay Kit | Beyotime | #C1056 |
| Cell Cycle and Apoptosis Analysis Kit | Beyotime | C1052 |
| RNAprep Pure Tissue Kit | TIANGEN | #DP431 |
| RNAprep Pure Cell/Bacteria Kit | TIANGEN | #4992235 |
| DNase I Kit | Sigma-Aldrich | #AMPD1-1KT |
| FastKing RT Kit (With gDNase) | TIANGEN | #KR116-02 |
| TransStart Green qPCR Super-Mix | TransGen Biotech | #AQ101-01 |
| One-step TUNEL cell apoptosis detection kit | Beyotime | #C1086 |
| ECL plus kit | Merck | #Cytiva RPN2106 |
| LiveBLAzer FRET-B/G Loading Kit | Invitrogen | #K1085 |
| Immunol Staining Fix Solution | Beyotime | #P0098 |
| TRITC Phalloidin | YESEN | #40734ES75 |
| DNA Damage Assay Kit by γ-H2AX Immunofluorescence | Beyotime | #C2035S |
| Trypsin | Beyotime | #C0209 |
| DNA Content Quantitation Assay | Solarbio | #CA1510 |
| Mouse TNFα ELISA Kit (High-sensitive) | Beyotime | #PT513 |
| Other | | |
| Flow cytometry | Beckman | N/A |
| FlowJo_V10 software | FlowJo | N/A |
| GraphPad Prism 8.01 | GraphPad | https://www.graphpad.com/ |
| Bacterial strains and plasmids used in this study | | |
| Bacterial strains and plasmids used in this study for strain construction: See Table S1. | | |
| Primers used in this study | | |
| Primers for strain construction: See Table S2. |  |  |
| Accession number of 16S rRNA gene sequencing | PRJNA1157218 | N/A |
| Accession number of RNA-seq libraries | PRJNA1157218 | N/A |

**References**

1. Simon R. *et al.* A broad host range mobilization system for in vivo genetic engineering: transposon mutagenesis in gram negative bacteria. *Nat Biotechnol* **1**, 784–791 (1983)
2. Wang, T. *et al.* Type VI Secretion System Transports Zn^2+^ to Combat Multiple Stresses and Host Immunity. *PLoS Pathog* **11**, e1005020 (2015)
3. Heeb, S., Blumer, C. & Haas, D. Regulatory RNA as mediator in GacA/RsmA-dependent global control of exoproduct formation in Pseudomonas fluorescens CHA0. *J Bacteriol* **184**, 1046-1056 (2002)
4. Zhao Y. *et al.* The NAIP-NLRC4 inflammasome in innate immune detection of bacterial flagellin and type III secretion apparatus. *Immunol Rev* **265(1)**, 85-102 (2015)
5. Hu, Y. *et al.* OmpR positively regulates urease expression to enhance acid survival of Yersinia pseudotuberculosis. *Microbiology* **155**, 2522-2531 (2009).
6. Karimova, G., Pidoux, J., Ullmann, A. & Ladant, D. A bacterial two-hybrid system based on a reconstituted signal transduction pathway. *Proc Natl Acad Sci U S A* **95**, 5752-5756 (1998).
7. Jiang, Y. *et al.* Multigene editing in the Escherichia coli genome via the CRISPR-Cas9 system. *Appl Environ Microbiol* **81**, 2506-2514 (2015)
8. Zhang, L. *et al.* The Catabolite Repressor/Activator Cra Is a Bridge Connecting Carbon Metabolism and Host Colonization in the Plant Drought Resistance-Promoting Bacterium Pantoea alhagi LTYR-11Z. *Appl Environ Microbiol* **84** (2018).
